# Supplementary material for: Methyl radical chemistry in non-oxidative methane activation over metal single sites
Source: Nat Commun. 2023 Sep 15;14:5716. doi: 10.1038/s41467-023-41192-y (PMC10504359; doi:10.1038/s41467-023-41192-y)
Supplement: Supplementary file 1 — Supplementary Information [file 41467_2023_41192_MOESM1_ESM.docx]

*Supplementary Information*

**Methyl radical chemistry in non-oxidative methane activation over metal single sites**

Xin Huang^1,†^, Daniel Eggart^2,†^, Gangqiang Qin^1,3,†^, Bidyut Bikash Sarma^4^, Abhijeet Gaur^2^, Jiuzhong Yang^5^, Yang Pan^5^, Mingrun Li^1^, Jianqi Hao^1^, Hongfei Yu^1^, Anna Zimina^4^, Xiaoguang Guo^1^, Jianping Xiao^1^, Jan-Dierk Grunwaldt^2,4,^*, Xiulian Pan^1,^*, Xinhe Bao^1,^*

^1^ State Key Laboratory of Catalysis, 2011-Collaborative Innovation Center of Chemistry for Energy Materials, Dalian Institute of Chemical Physics, Chinese Academy of Sciences, Dalian 116023, China

^2^ Institute for Chemical Technology and Polymer Chemistry, Karlsruhe Institute of Technology, Karlsruhe 76131, Germany

^3^ University of Chinese Academy of Sciences, Beijing 100049, China

^4^ Institute of Catalysis Research and Technology, Karlsruhe Institute of Technology, Eggenstein-Leopoldshafen 76344, Germany

^5^ National Synchrotron Radiation Laboratory, University of Science and Technology, Hefei 230029, China

^†^ These authors contributed equally: Xin Huang, Daniel Eggart, Gangqiang Qin

* Corresponding author. E-mail: grunwaldt@kit.edu; panxl@dicp.ac.cn; xhbao@dicp.ac.cn

**This file includes:**

- **Supplementary 42 Figures**

**Supplementary Fig. 1.** Non-oxidative methane conversion performances over the catalysts

**Supplementary Fig. 2.** Distribution of integral hydrocarbons and coke over 10 h reaction

**Supplementary Fig. 3.** Relative intensity of MDA products over 0.5Mo/MCM as a function of TOS

**Supplementary Fig. 4.** The photoionization efficiency of m/z = 15 signal

**Supplementary Fig. 5.** Relative intensity of methyl radical, C_2_H_4_, C_2_H_6_, and C_3_H_4_ as a function of reaction temperature over 0.5Mo/MCM catalyst

**Supplementary Fig. 6.** Distribution of C_2_-C_10_ products as a function of reaction temperature over 0.5Mo/MCM, detected by an online GC

**Supplementary Fig. 7.** XRD patterns of fresh and used xMo/MCM after reaction at 750 ℃ for 10 h

**Supplementary Fig. 8.** HAADF-STEM images

**Supplementary Fig. 9.** Magnitude of k^2^-weighted Fourier transforms recorded at Mo K-edge of samples

**Supplementary Fig. 10.** *Operando* XAS measurement procedure for samples

**Supplementary Fig. 11.** Effluents monitored by an online quadrupole MS during *operando* XAS measurement over 0.5Mo/MCM catalyst

**Supplementary Fig. 12.** Evolution of C_6_H_6_ (m/z = 78) with alternative CH_4_ pulses over *x*Mo/MCM catalysts and *ex situ* Mo K-edge XANES spectra of *x*Mo/MCM after 30 CH_4_ pulse reaction

**Supplementary Fig. 13.** k^2^-weighted Mo K-edge EXAFS spectra and the corresponding Fourier transforms (not corrected for phase-shift)

**Supplementary Fig. 14.** CO yield as a function of TOS over 0.5Mo/MCM catalyst

**Supplementary Fig. 15.** Periodic ^12^CH_4_/^13^CO switch pulse experiment, and periodic ^12^CH_4_/^13^CH_4_ switch pulse experiment over the 0.5Mo/MCM catalyst

**Supplementary Fig. 16.** FEFF 9-simulated XANES spectra of specific single Mo site structures with various Mo-CH_x_ and Mo-O paths

**Supplementary Fig. 17.** The free energy of different Mo single sites (O=Mo-CH_x_)

**Supplementary Fig. 18.** The XANES linear combination fitting of the simulated O=Mo=O and O=Mo-CH_2_

**Supplementary Fig. 19.** Structural transformation of single Mo sites during the MDA reaction

**Supplementary Fig. 20.** SVUV-PIMS spectra over 0.5Mo/MCM

**Supplementary Fig. 21.** Ethane dehydrogenation in the absence of catalyst

**Supplementary Fig. 22.** MDA reaction over 0.5Mo/ZSM

**Supplementary Fig. 23.** NH_3_-TPD profiles of fresh 0.5Mo/MCM, 0.5Mo/ZSM and 0.5Mo/SiO_2_

**Supplementary Fig. 24.** HAADF-STEM images

**Supplementary Fig. 25.** *Operando* Mo K-edge XANES spectra over 0.5Mo/ZSM during MDA reaction

**Supplementary Fig. 26.** k^2^-weighted Mo K-edge EXAFS spectra and the corresponding Fourier transform of 0.5Mo/ZSM(CH_4_) after MDA reaction

**Supplementary Fig. 27.** Relative concentration of gas-phase methyl radicals over 0.5Mo/ZSM and 0.5Mo/MCM catalysts

**Supplementary Fig. 28.** Normalized concentration of methyl radicals as a function of Mo loadings over *x*Mo/MCM and 0.5Mo/SiO_2_

**Supplementary Fig. 29.** Nitrogen adsorption-desorption isotherms and corresponding pore size distributions for fresh 0.5Mo/SiO_2_.

**Supplementary Fig. 30.** TEM and HAADF-STEM images of fresh 0.5Mo/SiO_2_

**Supplementary Fig. 31.** k^2^-weighted Mo K-edge EXAFS spectra and the corresponding Fourier transforms

**Supplementary Fig. 32.** Distribution of cumulative hydrocarbon products of 0.5Mo/SiO_2_ in comparison to 0.5Mo/MCM

**Supplementary Fig. 33.** HAADF-STEM image of used 0.5Mo/SiO_2_ after fixed-bed test at 750 ℃ for 10 h

**Supplementary Fig. 34.** XRD patterns of fresh 0.5Mo/SiO_2_ and used 0.5Mo/SiO_2_ after fixed-bed test at 750 ℃

**Supplementary Fig. 35.** *Operando* Mo K-edge XANES spectra during MDA reaction over 4Mo/MCM at 750 ℃

**Supplementary Fig. 36.** HAADF-STEM image and the elemental maps (Mo, O, C, Si, Al) of used 4Mo/MCM catalysts after MDA reaction at 750 ℃ for 10 h

**Supplementary Fig. 37.** HRTEM images of used 2Mo/MCM and 4Mo/MCM catalysts after MDA reaction at 750 ℃ for 10 h

**Supplementary Fig. 38.** The free energy diagrams (at 750 ℃) of CH_4_ conversion towards CH_3_CH_3_(g) on Mo_2_C{101}

**Supplementary Fig. 39.** Ethane aromatization and ethylene aromatization over pure H-MCM-22

**Supplementary Fig. 40.** View and key equipment of the setup used for the *operando* XAS measurement

**Supplementary Fig. 41.** k^2^-weighted Mo K-edge EXAFS spectra and the corresponding Fourier transforms

**Supplementary Fig. 42.** Schematic of the catalytic reactor with the SVUV-PIMS system and measured centerline temperature profiles

- **Supplementary 4 Tables**

**Supplementary Table 1.** ICP results of Mo-bearing catalysts

**Supplementary Table 2.** EXAFS fitting for 0.5Mo/MCM acquired EXAFS scan at 40 ℃ after He calcination and MDA reaction during *operando* XAS experiment

**Supplementary Table 3.** EXAFS fitting for 0.5Mo/ZSM acquired EXAFS scan at 40 ℃ after MDA reaction during *operando* XAS experiment

**Supplementary Table 4.** Structural parameters extracted from the Mo K-edge EXAFS fitting

- **Supplementary References**


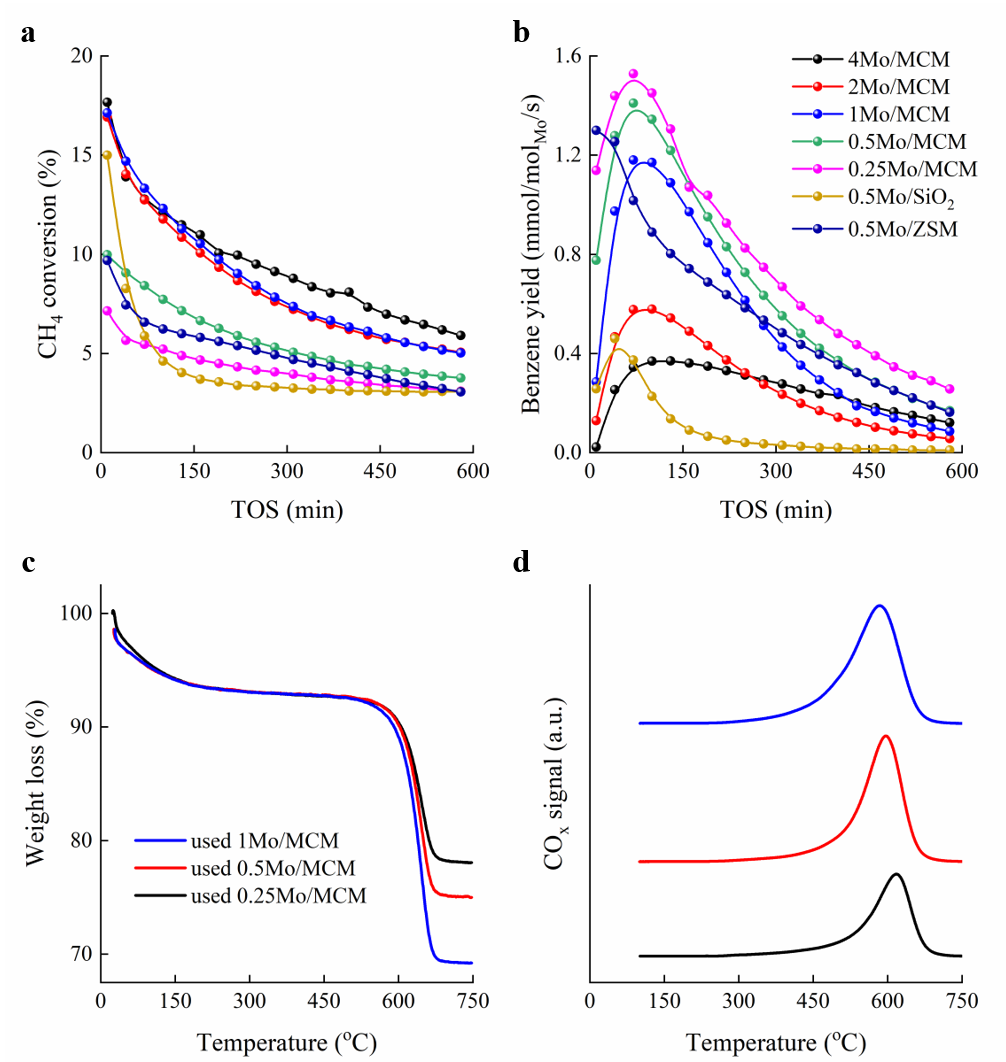


# Supplementary Fig. 1 | Non-oxidative methane conversion performances over the catalysts. **a**, CH_4_ conversion and **b**, benzene yield as a function of time of stream (TOS), **c**, TG and **d**, TPO profiles of used *x*Mo/MCM catalysts after reaction at 750 ℃ for 10 h, showing that the total amount of carbonaceous deposition increases with the Mo loading. Reaction conditions: 750 ℃, 0.1 MPa, 1.5 L/(g_cat_·h), and CH_4_:N_2_ = 9:1.





# Supplementary Fig. 2 | Distribution of integral hydrocarbons and coke over 10 h reaction. Reaction conditions: 750 ℃, 0.1 MPa, 1.5 L/(g_cat_·h), and CH_4_:N_2_ = 9:1.


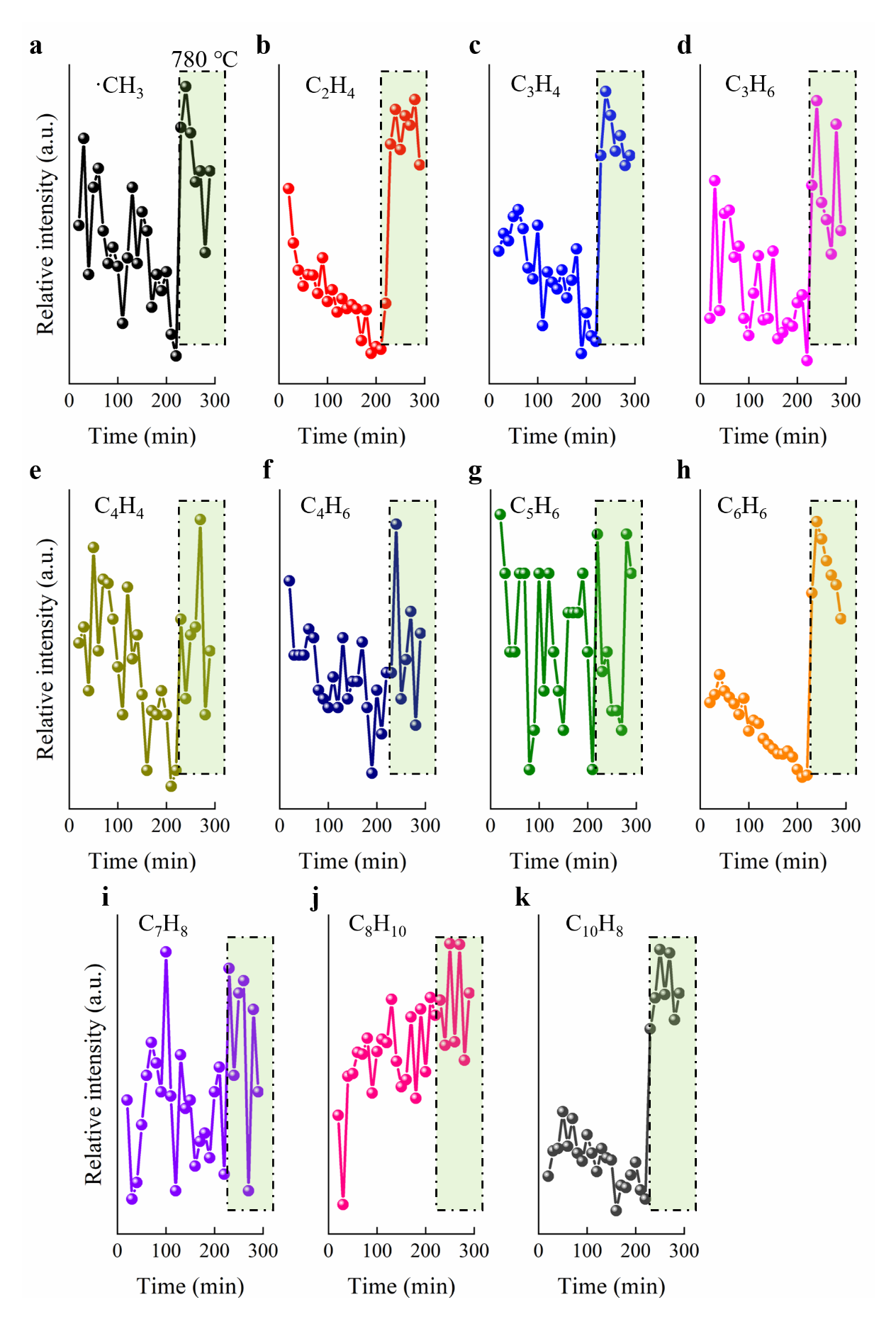


# Supplementary Fig. 3 | Relative intensity of MDA products over 0.5Mo/MCM as a function of TOS. Conditions: photon energy 10.6 eV, 750 ℃ (0-220 min)/780 ℃ (230-300 min), 0.47 kPa, and CH_4_ GHSV 12 L/(g_cat_·h).





# Supplementary Fig. 4 | The photoionization efficiency of m/z = 15 signal. Reaction conditions: 750 ℃, 0.47 kPa, methane GHSV 12 L/(g_cat_·h), 1Mo/MCM.





# Supplementary Fig. 5 | Relative intensity of methyl radical, C_2_H_4_, C_2_H_6_, and C_3_H_4_ as a function of reaction temperature over 0.5Mo/MCM catalyst. Reaction condition: photon energy 10.6 eV/12.0 eV, pressure 0.47 kPa, and CH_4_ GHSV 12 L/(g_cat_·h).

# Supplementary Fig. 6 | Distribution of C_2_-C_10_ products as a function of reaction temperature over 0.5Mo/MCM, detected by an online GC. Reaction temperature: 0.1 MPa, CH_4_:N_2_ = 9:1, and 1500 mL/(g·h). Note that the temperature was ramped at 5 ℃/min from 600 to 780 ℃. Each reaction temperature was kept for 15 min, acquiring the GC data at 10 min. C_2_H_4_ and C_2_H_6_ simultaneously start to form at *ca.* 630 ℃. Very little C_3_ hydrocarbons are observed starting from 710 ℃. With the temperature increasing to above 750 ℃, aromatics start to be detected and the concentration significantly increases at 760 ℃.





# Supplementary Fig. 7 | XRD patterns of fresh and used *x*Mo/MCM after reaction at 750 ℃ for 10 h (**○** represents diffraction peaks of MCM-22 zeolite).


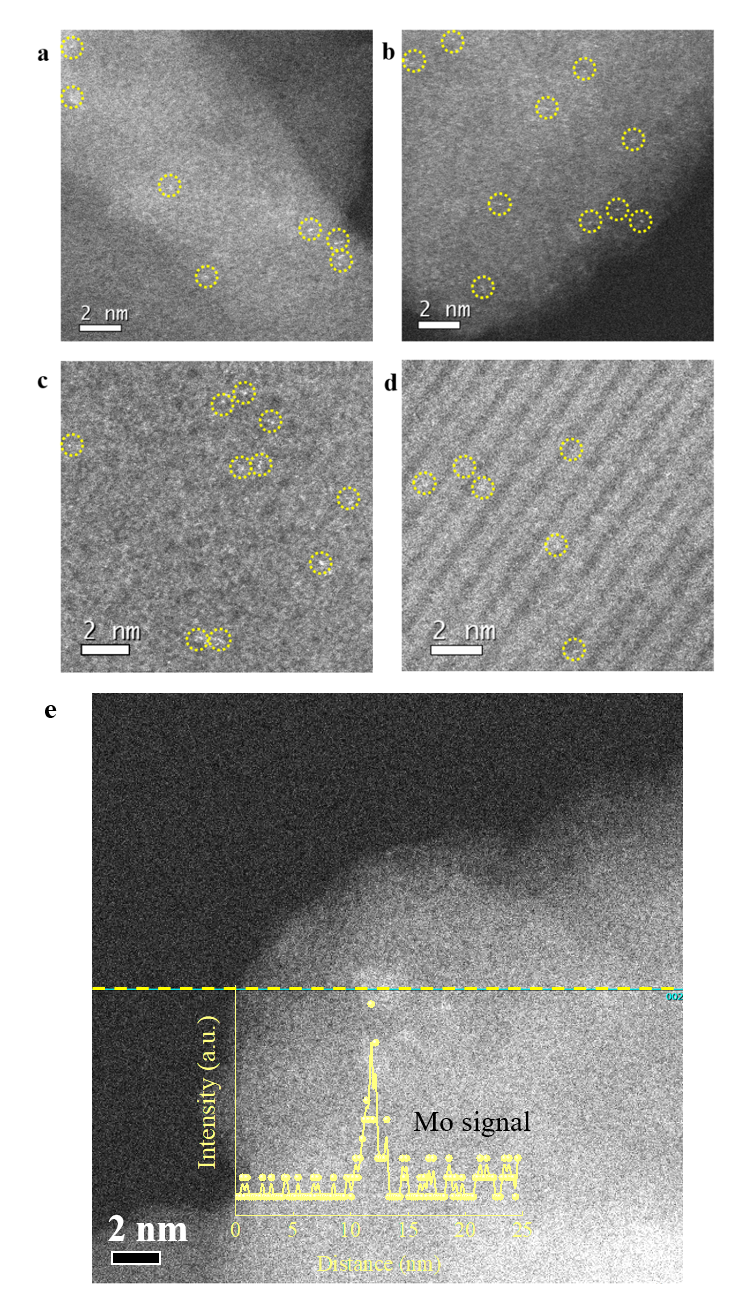


# Supplementary Fig. 8 | HAADF-STEM images. **a**, **e,** Fresh 0.25Mo/MCM (with the inset showing an EDX line scanning of Mo), **b**, used 0.25Mo/MCM, **c,** fresh 1Mo/MCM. **d**, used 1Mo/MCM. Reaction conditions: 750 ℃ for 10 h.


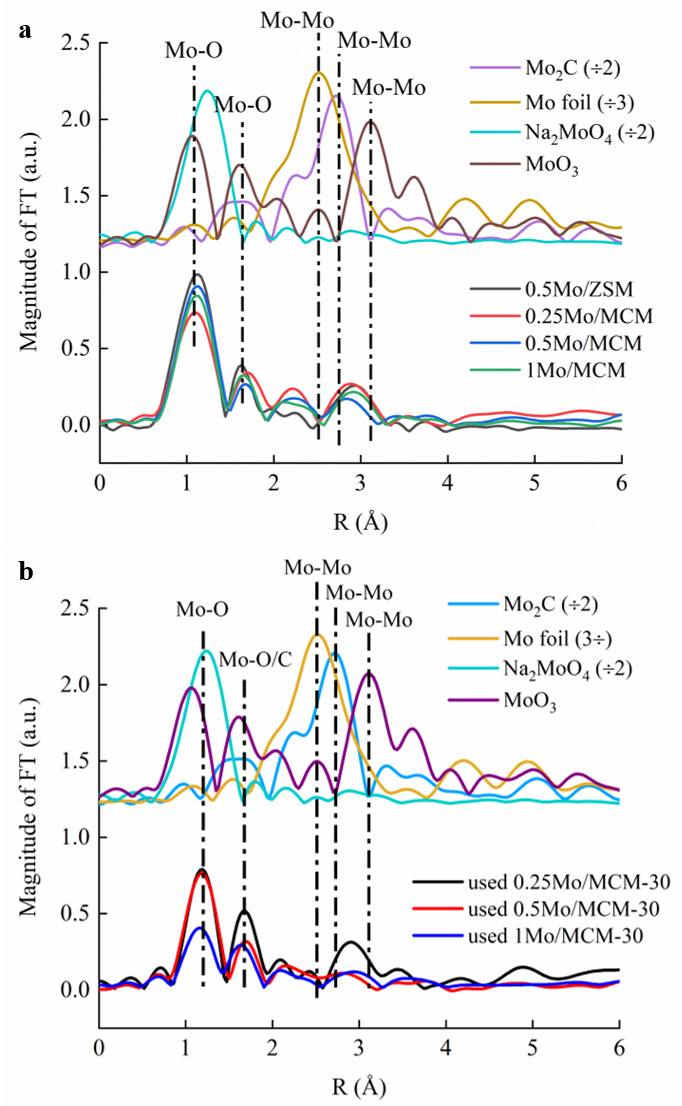


# Supplementary Fig. 9 | Magnitude of k^2^-weighted Fourier transforms recorded at Mo K-edge of **a**, fresh *x*Mo/MCM and 0.5Mo/ZSM, and **b**, used *x*Mo/MCM after 30 CH_4_ pulse reaction (Conditions: pulse reaction with 5 mL 90 vol.%CH_4_/He and 30 mL/min Ar flow at 750 ℃), along with Mo foil, Mo_2_C, Na_2_MoO_4_, and MoO_3_ references. Note that there is an increase of Mo-Mo distance starting from Mo foil, *via* Mo carbide to Mo oxide.


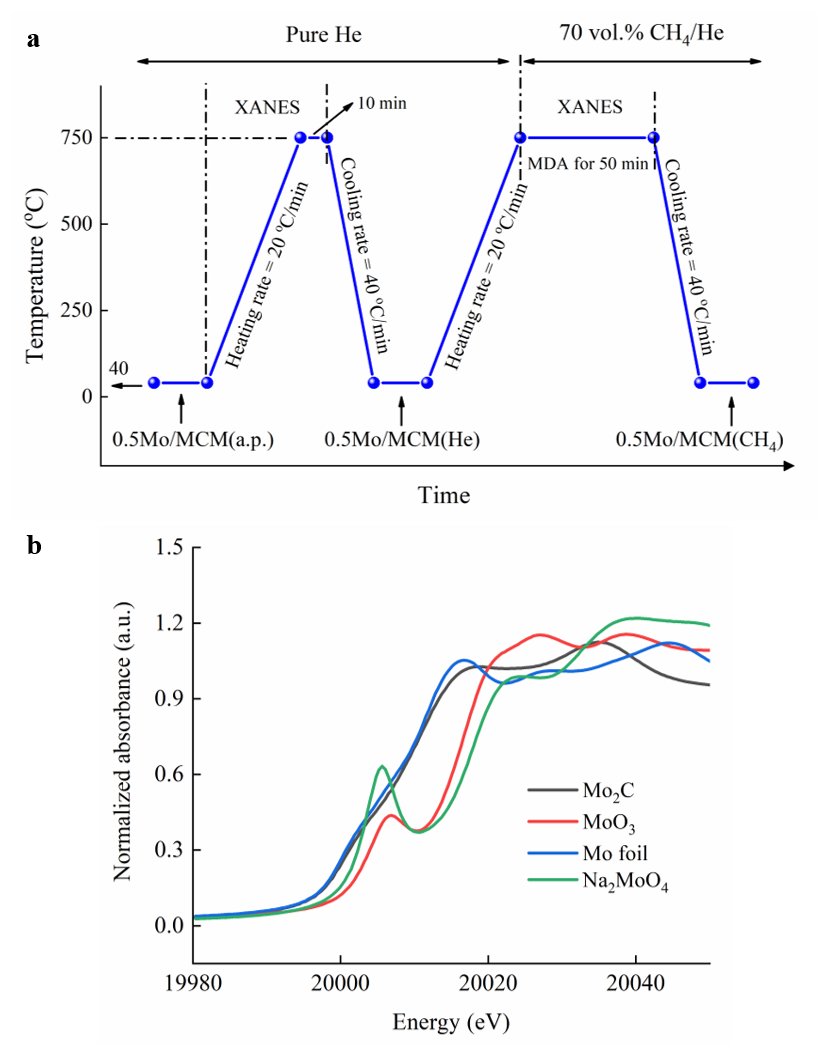


# Supplementary Fig. 10 | **a**, *Operando* XAS measurement procedure for 0.5Mo/MCM, 0.5Mo/SiO_2_, and 4Mo/MCM, and **b**, Mo K-edge XANES spectra of Mo_2_C, MoO_3_, Mo foil, and Na_2_MoO_4_ references.





# Supplementary Fig. 11 | Effluents monitored by an online quadrupole MS during *operando* XAS measurement over 0.5Mo/MCM catalyst. Reaction conditions: 750 ℃, 0.1 MPa, 70 vol.%CH_4_/He.

# Supplementary Fig. 12 | **a, c, e,** Evolution of C_6_H_6_ (m/z = 78) with alternative CH_4_ pulses over *x*Mo/MCM catalysts, and **b, d, f,** *ex situ* Mo K-edge XANES spectra of *x*Mo/MCM after 30 CH_4_ pulse reaction. Conditions: 200 mg, pulse reaction with 5 mL 90 vol.%CH_4_/He and 30 mL/min Ar flow at 750 ℃.


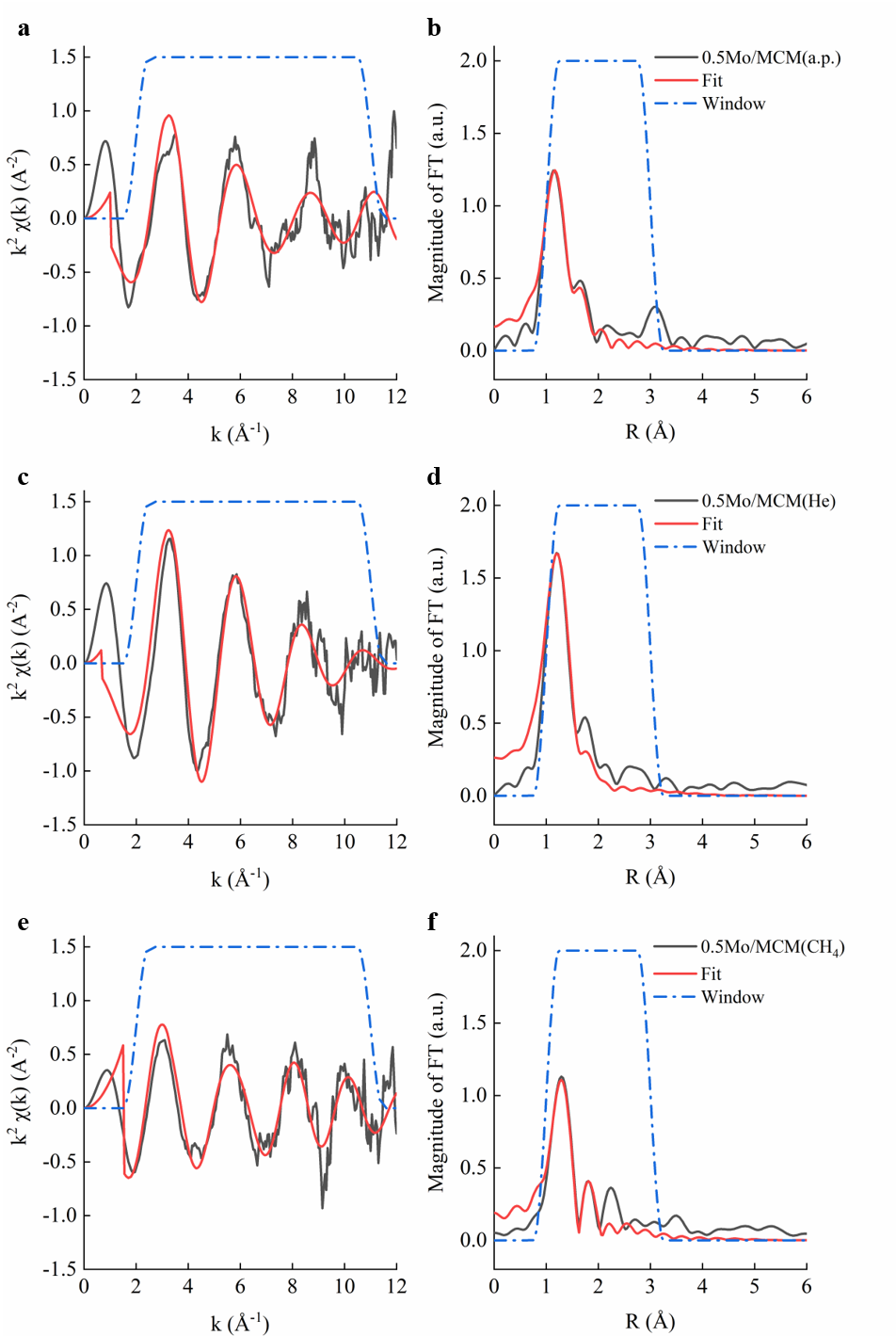


# Supplementary Fig. 13 | k^2^-weighted Mo K-edge EXAFS spectra and the corresponding Fourier transforms (not corrected for phase-shift). **a, b**, Fresh 0.5Mo/MCM(a.p.), **c, d**, 0.5Mo/MCM(He) being heated in He, and **e, f**, 0.5Mo/MCM(CH_4_) after MDA reaction. All spectra were collected at 40 ℃ in the *operando* cell.





# Supplementary Fig. 14 | CO yield as a function of TOS over 0.5Mo/MCM catalyst. Reaction conditions: 750 ℃, 0.1 MPa, 1.5 L/(g_cat_·h), and CH_4_:N_2_ = 9:1. CO formation is detected during the initial stage of MDA reaction, indicating that the oxygen from the Mo-oxo species is consumed due to reduction. It was estimated that ~20% Mo species have been reduced according to the CO yield determined by GC. However, it is not clear yet the role of the remaining O=Mo=O sites in the reaction, which should be further investigated in the future.


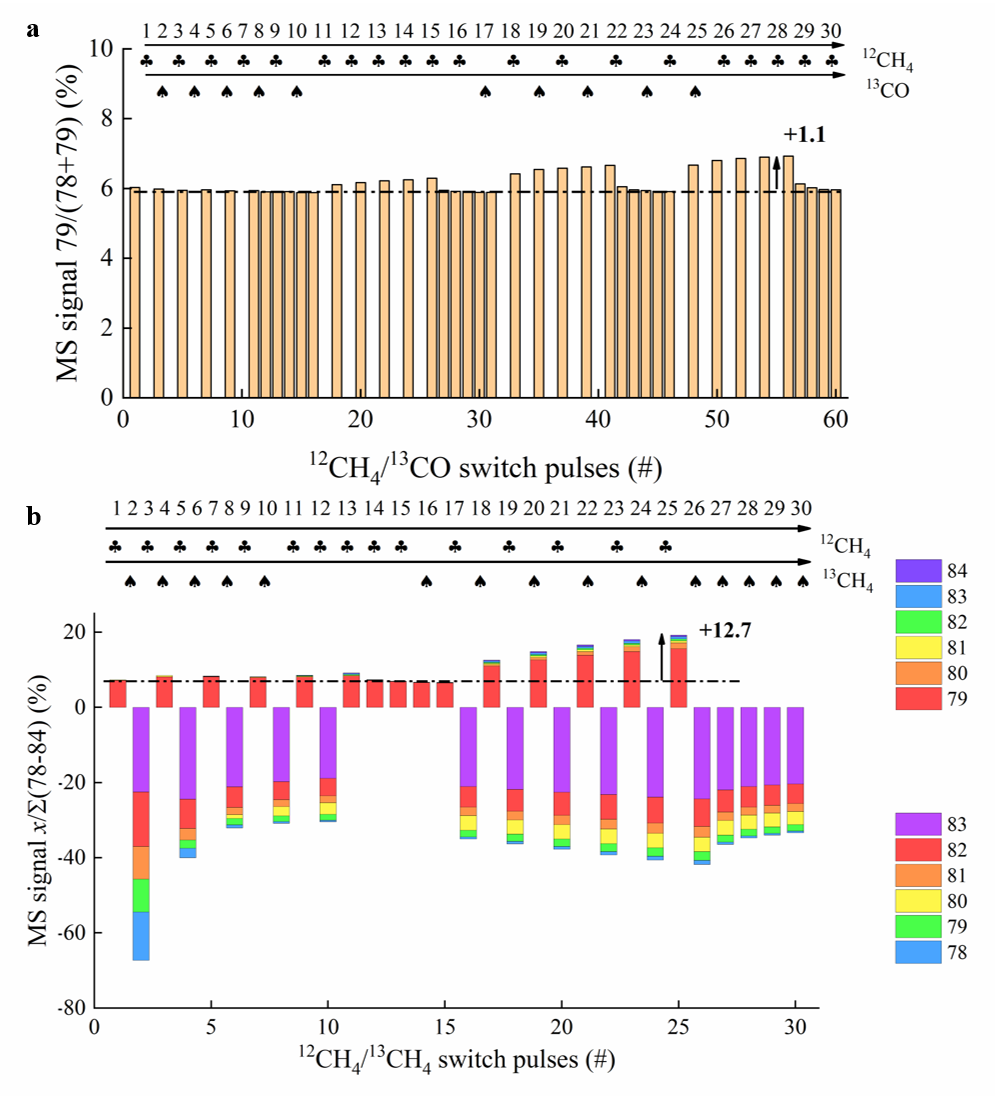


# Supplementary Fig. 15 | **a**, Periodic ^12^CH_4_/^13^CO switch pulse experiment, and **b**, periodic ^12^CH_4_/^13^CH_4_ switch pulse experiment over the 0.5Mo/MCM catalyst. Reaction conditions: 200 mg catalyst, 750 ℃, 0.1 MPa, carrier gas: 15 mL/min of Ar, pulse gas A: ^12^CH_4_, pulse gas B: 2.5 vol.% ^13^C^18^O/He, pulse gas C: ^13^CH_4_, pulse is allowed 5 mL every 6 min. No hydroformylation products containing oxygen were detected during the periodic ^12^CH_4_/^13^CO switch pulse reaction, suggesting that there may not be hydroformylation reaction.


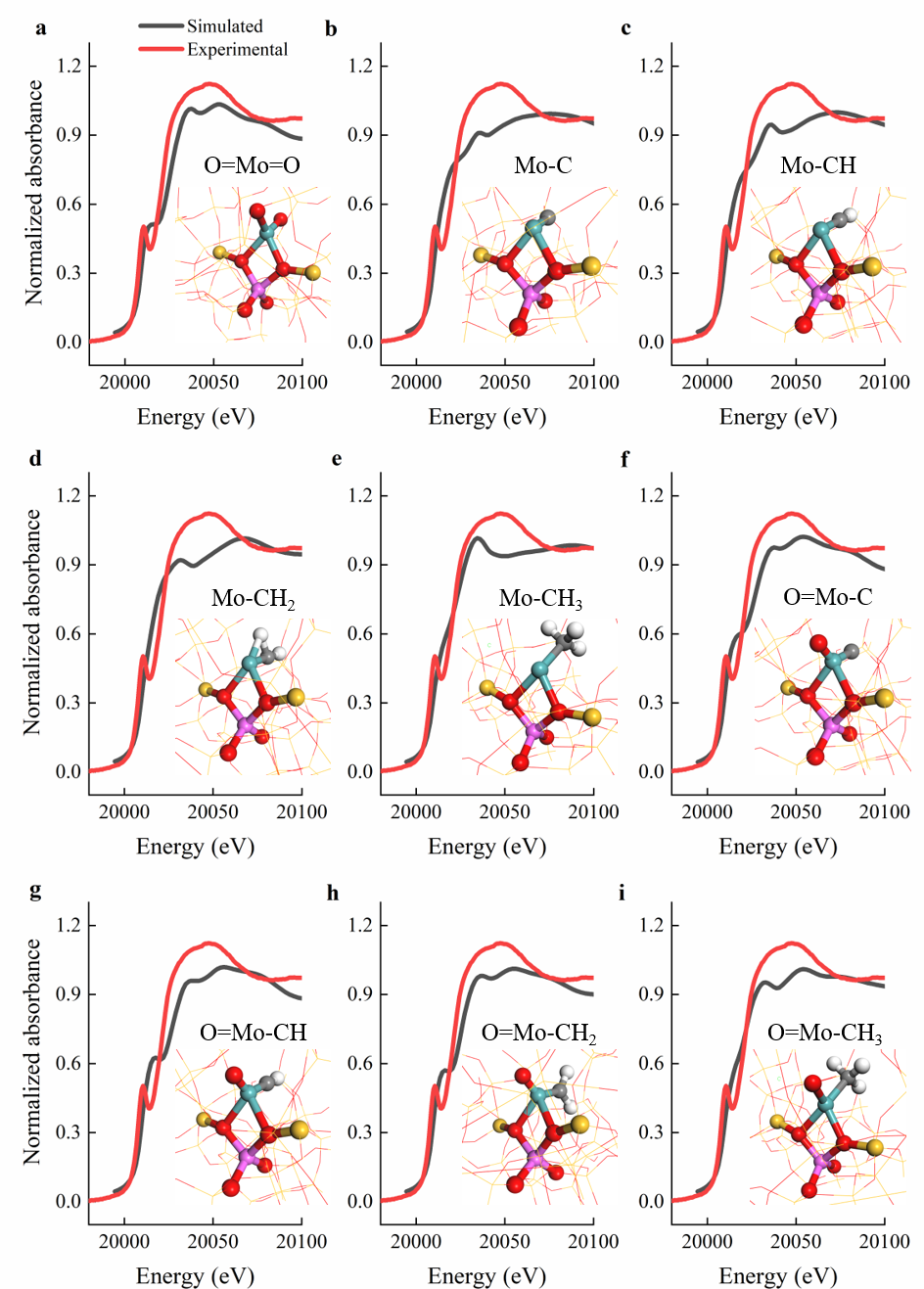


# Supplementary Fig. 16 | FEFF 9-simulated XANES spectra of specific single Mo site structures with various Mo-CH_x_ and Mo-O paths using the FEFF 9.6.4 code^1^. $S_{0}^{2}$, amplitude reduction factor was set to 1 and the core-hole was treated with random phase approximation. The self-consistent field (SCF) parameters were rfms = 4, lfms1 = 0, nscmt = 100, ca = 0.2, nmix = 1 and the XANES parameters were xkmax = 4, xkstep = 0.07, vixan = 0. The LDOS card was added for density of states calculation with an energy range of -20 to 30 eV with a Lorentzian broadening with half-width of 0.1 eV. The radii considered for full multiple scattering (FMS) and SCF calculations were 5 Å.


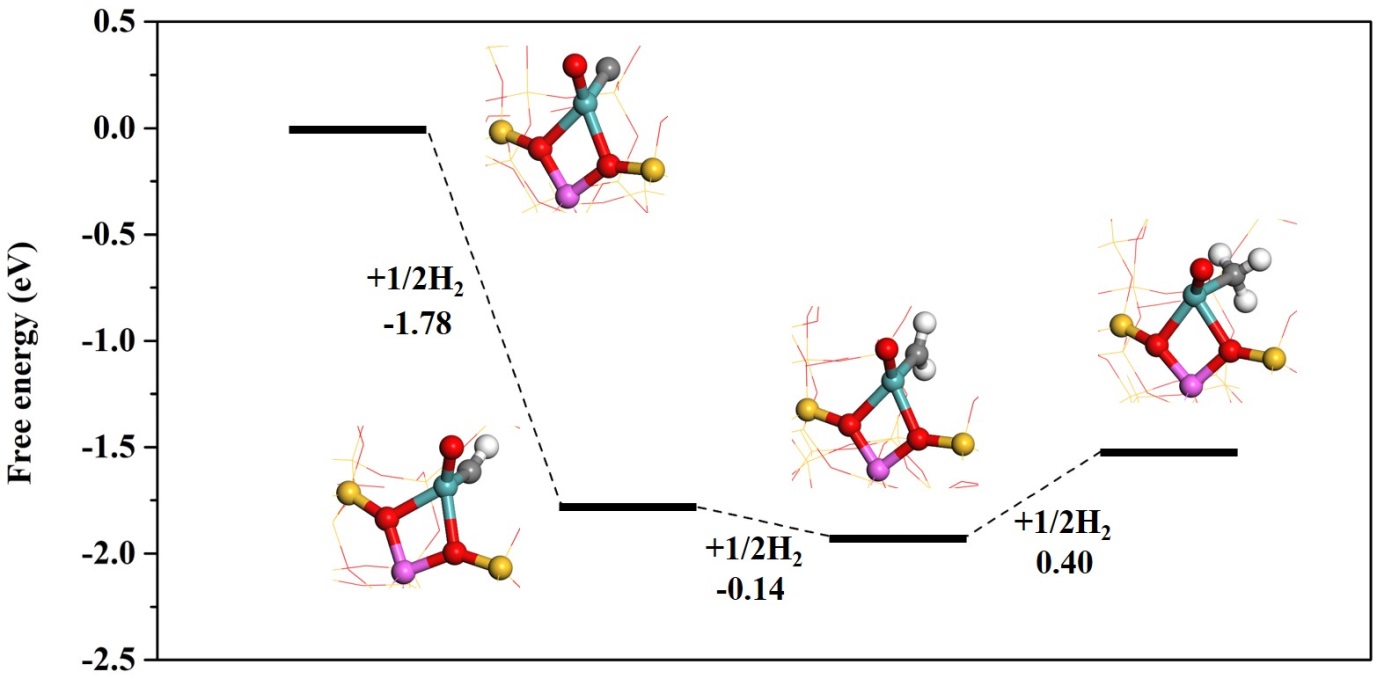


# Supplementary Fig. 17 | The free energy of different Mo single sites (O=Mo-CH_x_), indicating that O=Mo-CH_2_ is thermodynamically the most stable structure. The white, grey, red, pink, yellow and green balls are H, C, O, Al, Si and Mo atoms, respectively.





# Supplementary Fig. 18 | The XANES linear combination fitting of the simulated O=Mo=O and O=Mo-CH_2_


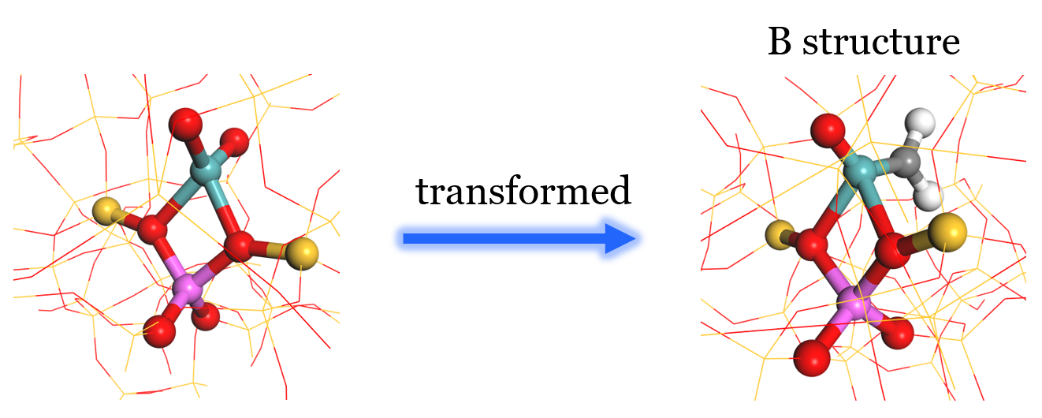


# Supplementary Fig. 19 | Structural transformation of single Mo sites during the MDA reaction. The white, gray, red, pink, yellow, and green balls represent H, C, O, Al, Si, Mo atoms, respectively.


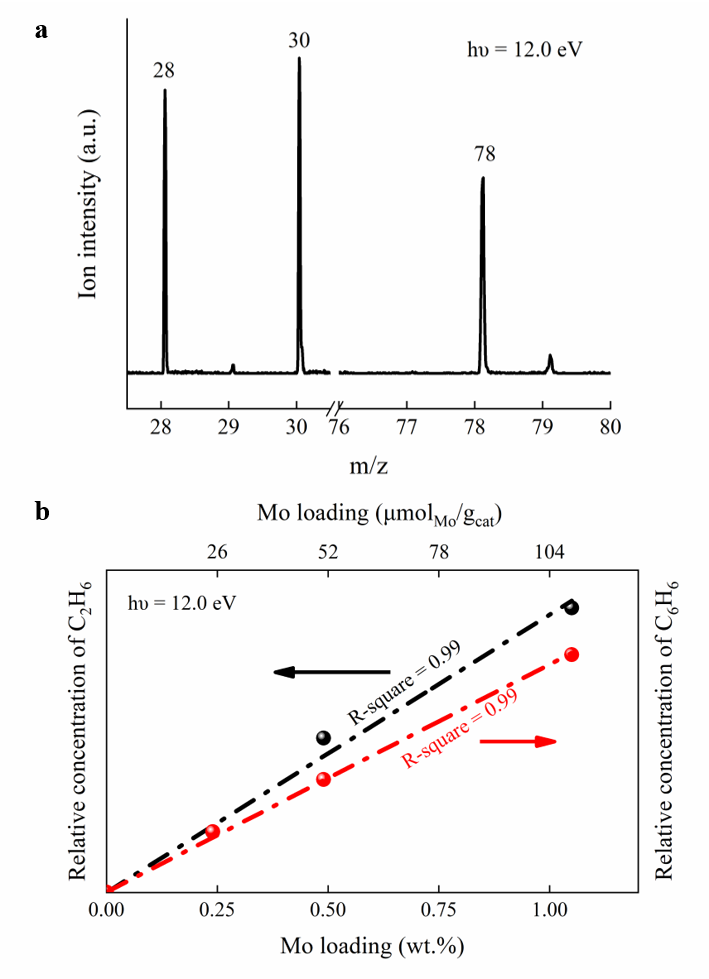


# Supplementary Fig. 20 | SVUV-PIMS spectra over 0.5Mo/MCM. Conditions: 750 ℃, 0.47 kPa, CH_4_ GHSV 12 L/(g_cat_·h), TOS = 40 min, and photon energy of 12.0 eV.





# Supplementary Fig. 21 | Ethane dehydrogenation in the absence of catalyst (Reaction conditions: 750 ℃, 0.1 MPa, C_2_H_6_:N_2_ = 1:9, flow rate of feed gas of 10 mL/min).





# Supplementary Fig. 22 | MDA reaction over 0.5Mo/ZSM. Reaction conditions: 750 ℃, 0.1 MPa, 1.5 L/(g_cat_·h), and CH_4_:N_2_ = 9:1.





# Supplementary Fig. 23 | NH_3_-TPD profiles of fresh 0.5Mo/MCM, 0.5Mo/ZSM and 0.5Mo/SiO_2_.


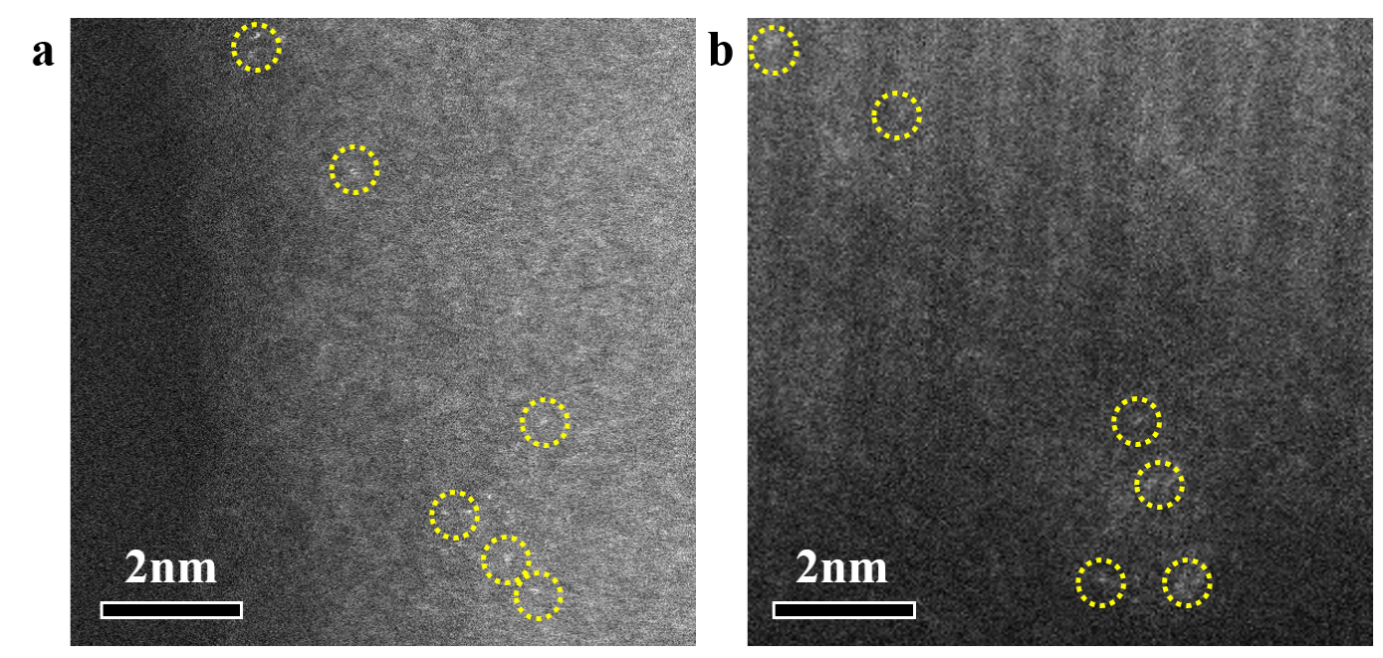


# Supplementary Fig. 24 | HAADF-STEM images. **a**, Fresh 0.5Mo/ZSM and **b**, used 0.5Mo/ZSM after reaction at 750 ℃ for 10 h.





# Supplementary Fig. 25 | *Operando* Mo K-edge XANES spectra over 0.5Mo/ZSM during MDA reaction at 750 ℃ and 0.1 MPa.





# Supplementary Fig. 26 | **a**, k^2^-weighted Mo K-edge EXAFS spectra and **b**, the corresponding Fourier transform of 0.5Mo/ZSM(CH_4_) after MDA reaction. The spectra were collected at 40 ℃ in the *operando* XAS cell.





# Supplementary Fig. 27 | Relative concentration of gas-phase methyl radicals over 0.5Mo/ZSM and 0.5Mo/MCM catalysts. Reaction: 750 ℃, 0.47 kPa, CH_4_ GHSV 12 L/(g_cat_⋅h).


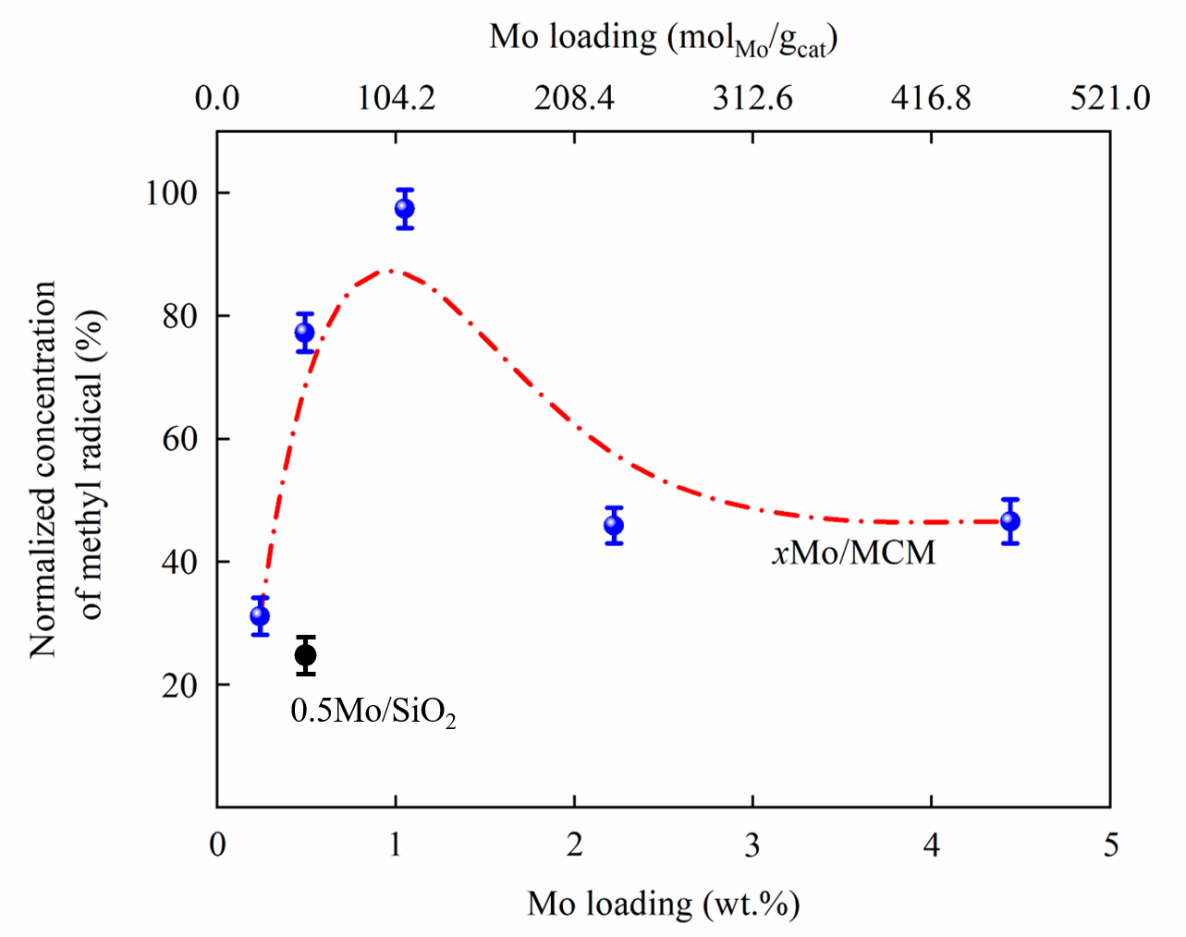


# Supplementary Fig. 28 | Normalized concentration of methyl radicals as a function of Mo loadings over *x*Mo/MCM and 0.5Mo/SiO_2_. Reaction conditions: 750 ℃, 0.47 kPa, CH_4_ GHSV 12 L/(g_cat_·h).





# Supplementary Fig. 29 | **a**, Nitrogen adsorption-desorption isotherms and **b**, corresponding pore size distributions for fresh 0.5Mo/SiO_2_.


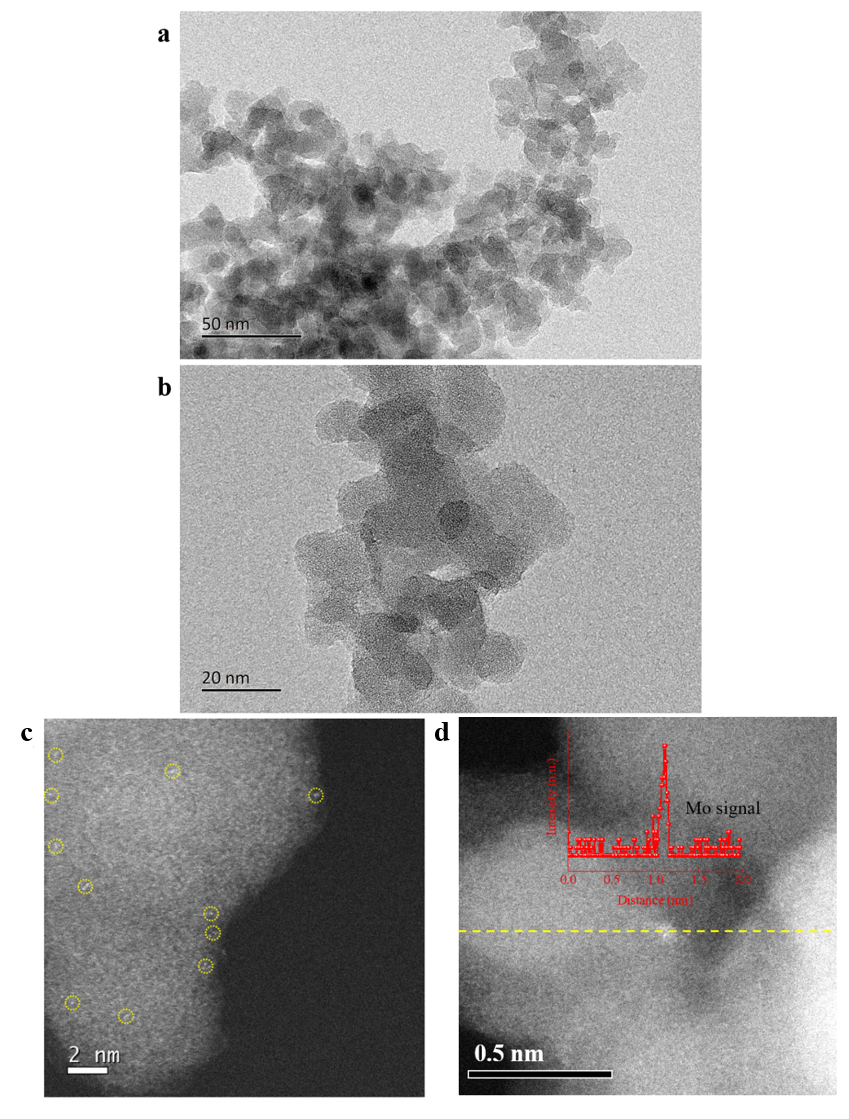


# Supplementary Fig. 30 | **a, b,** TEM, and **c, d,** HAADF-STEM images of fresh 0.5Mo/SiO_2_. The inserted yellow line shows the EDX line scanning of Mo element.


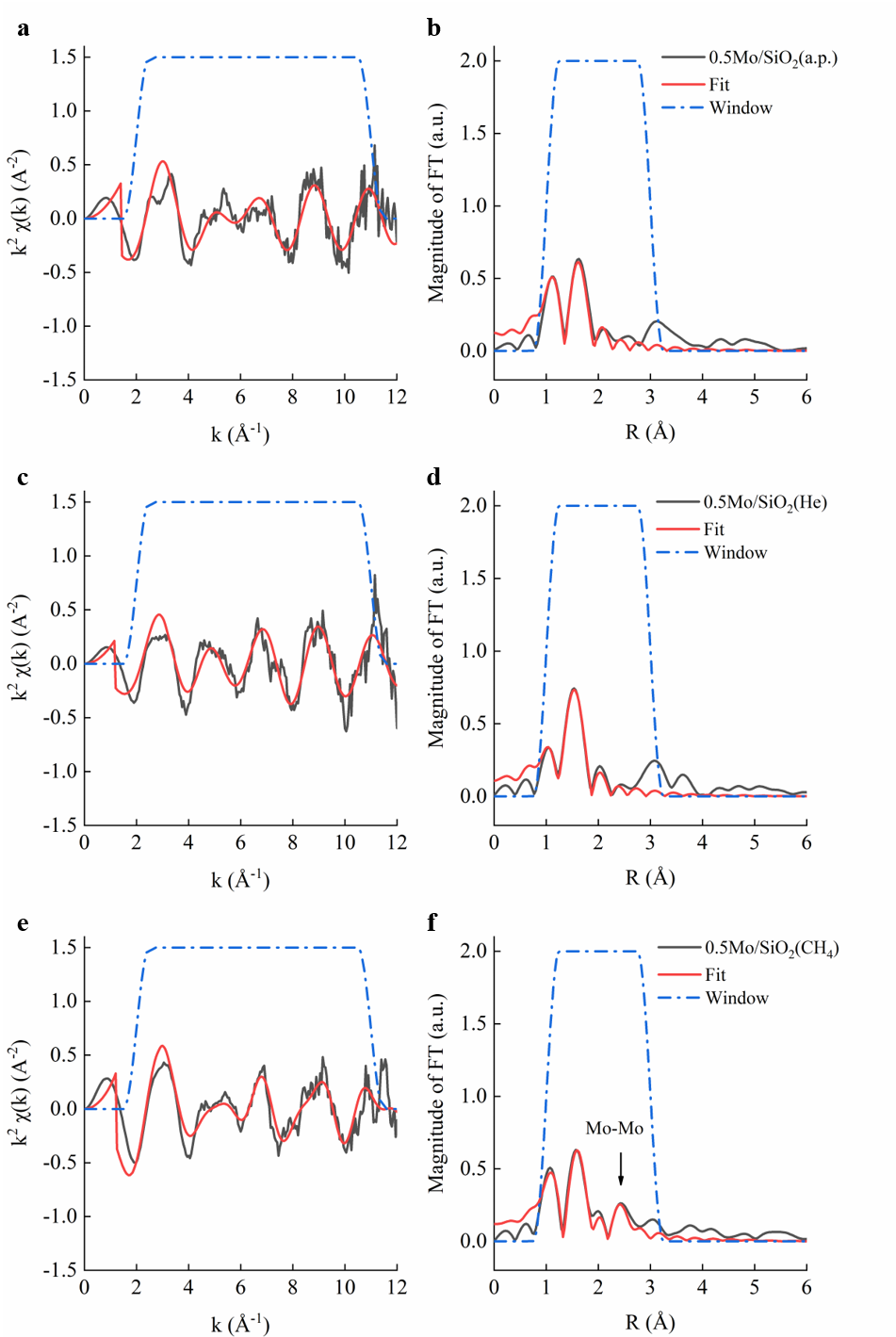


# Supplementary Fig. 31 | k^2^-weighted Mo K-edge EXAFS spectra and the corresponding Fourier transforms. **a, b**, Fresh 0.5Mo/SiO_2_(a.p.), **c, d**, 0.5Mo/SiO_2_(He) after He calcination, and **e, f**, 0.5Mo/SiO_2_(CH_4_) after MDA reaction. All spectra were collected at 40 ℃ in the *operando* XAS cell.





# Supplementary Fig. 32 | Distribution of cumulative hydrocarbon products of 0.5Mo/SiO_2_ in comparison to 0.5Mo/MCM. Reaction conditions: 750 ℃, CH_4_:N_2_ = 9:1, 0.1 MPa, 1.5 L/(g_cat_·h) and data obtained after 10 h on stream.


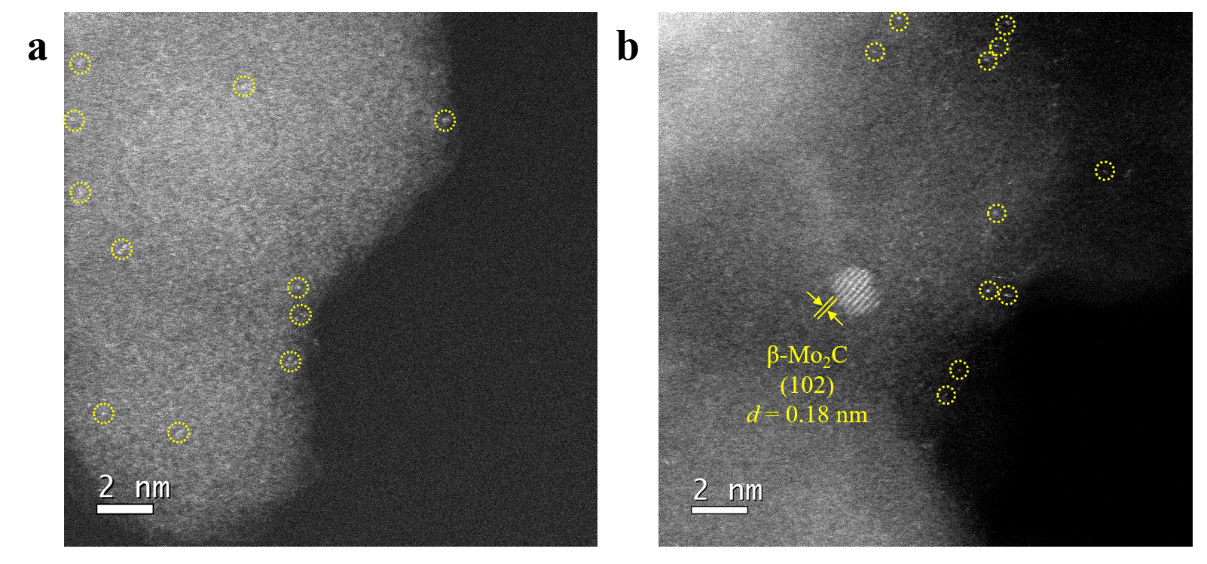


# Supplementary Fig. 33 | HAADF-STEM image of used 0.5Mo/SiO_2_ after fixed-bed test at 750 ℃ for 10 h.





# Supplementary Fig. 34 | XRD patterns of fresh 0.5Mo/SiO_2_ and used 0.5Mo/SiO_2_ after fixed-bed test at 750 ℃.


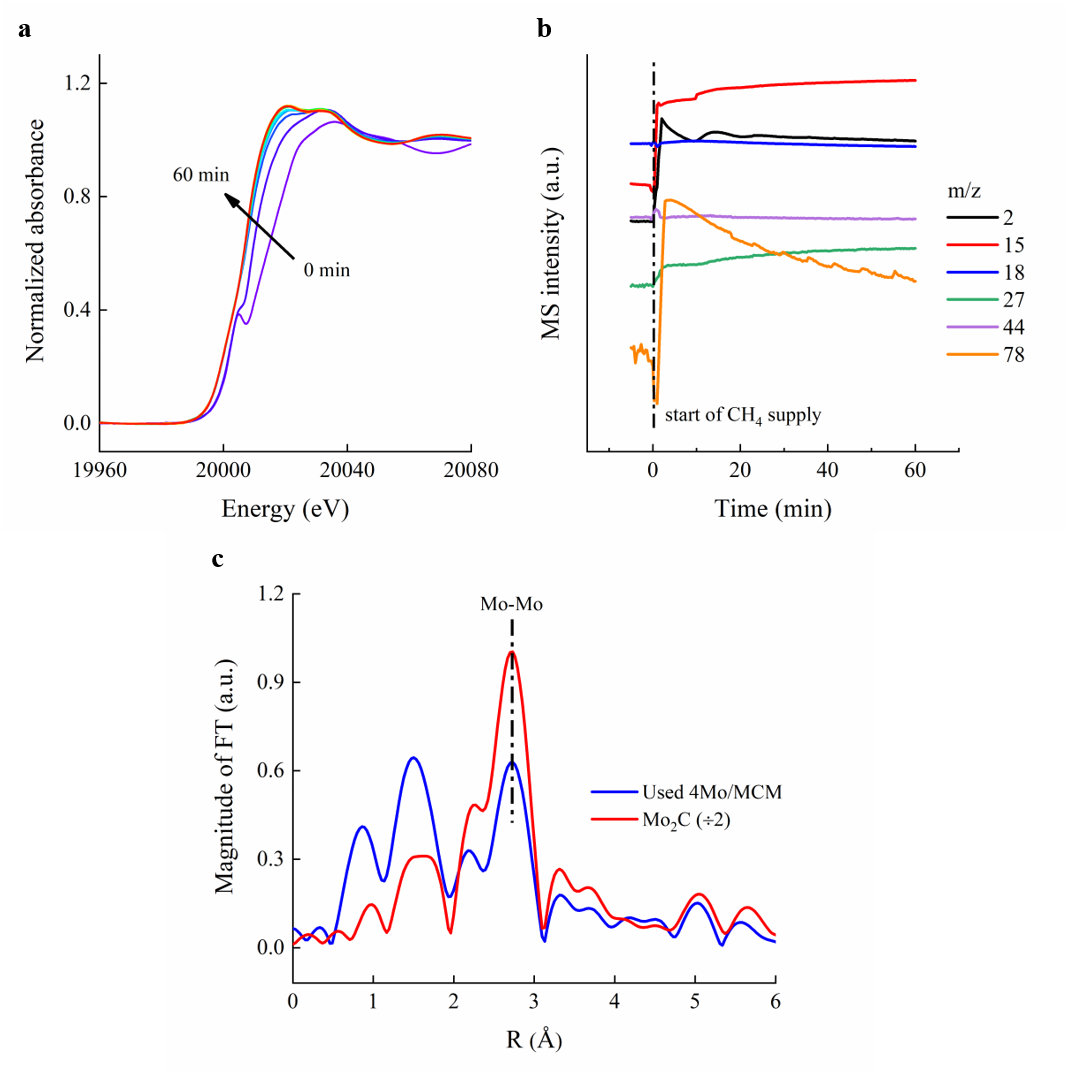


# Supplementary Fig. 35 | **a**, *Operando* Mo K-edge XANES spectra during MDA reaction over 4Mo/MCM at 750 ℃, **b**, the effluents were monitored by an online quadrupole MS during *operando* XAS measurement, **c**, magnitude of k^2^-weighted Fourier transform from EXAFS recorded at Mo K-edge of 4Mo/MCM(CH_4_) and Mo_2_C reference.


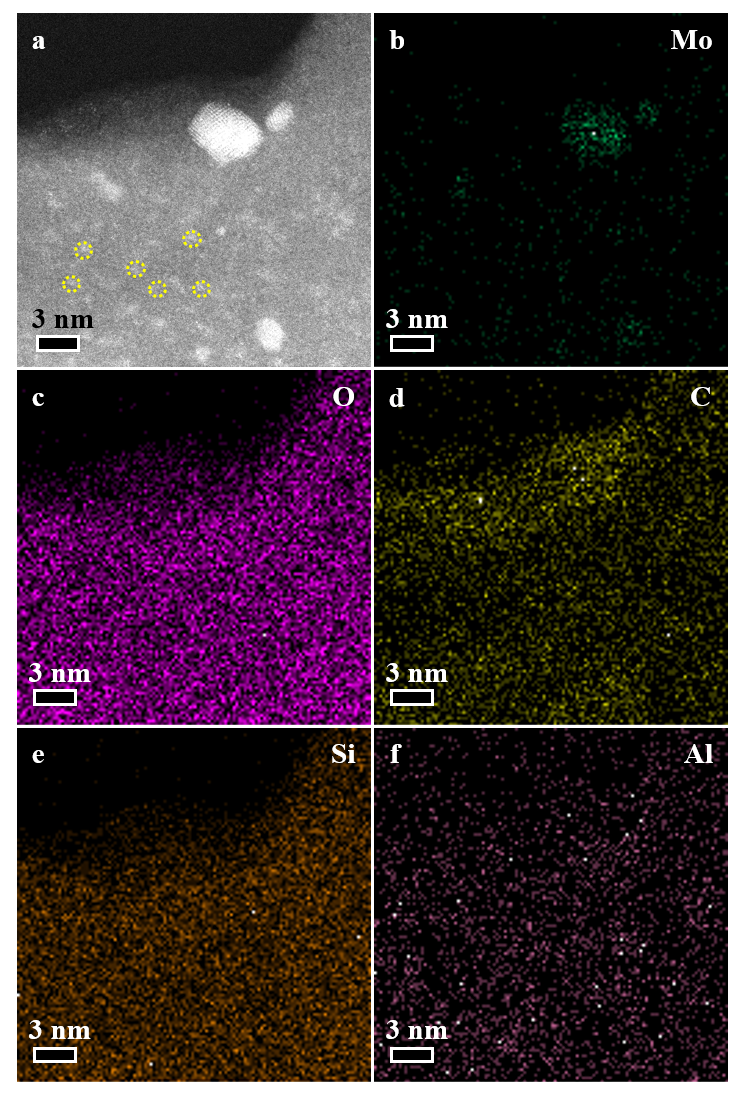


# Supplementary Fig. 36 | HAADF-STEM image (Note that the yellow circles highlight Mo single sites) and the elemental maps (Mo, O, C, Si, Al) of used 4Mo/MCM catalysts after MDA reaction at 750 ℃ for 10 h.


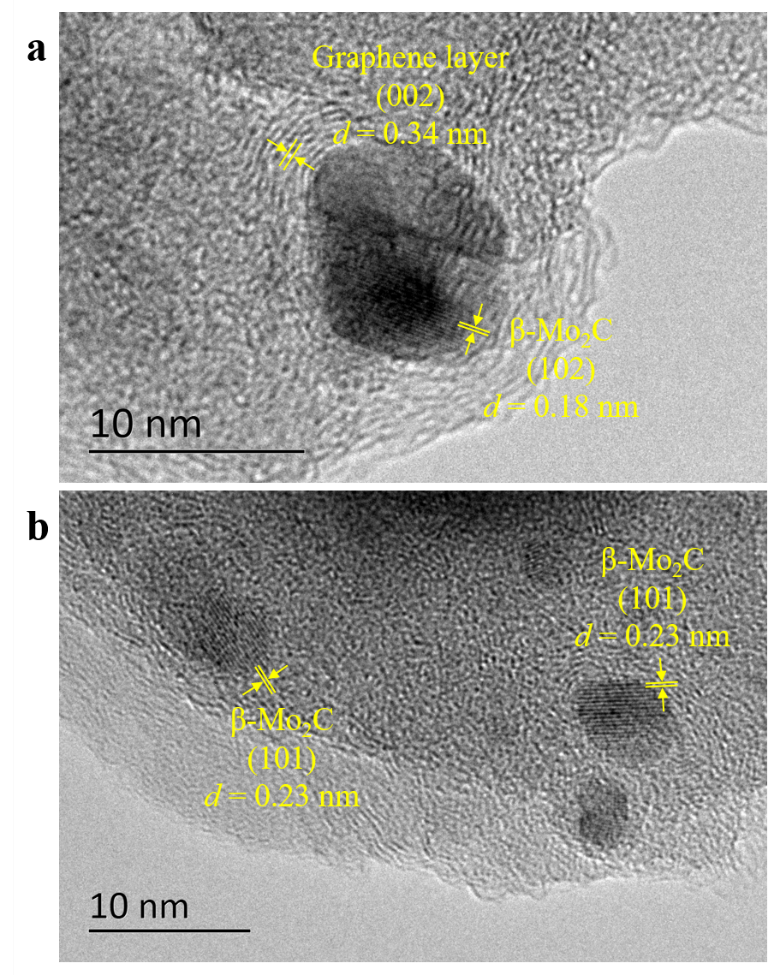


# Supplementary Fig. 37 | HRTEM images of **a**, used 2Mo/MCM and **b**, used 4Mo/MCM catalysts after MDA reaction at 750 ℃ for 10 h.


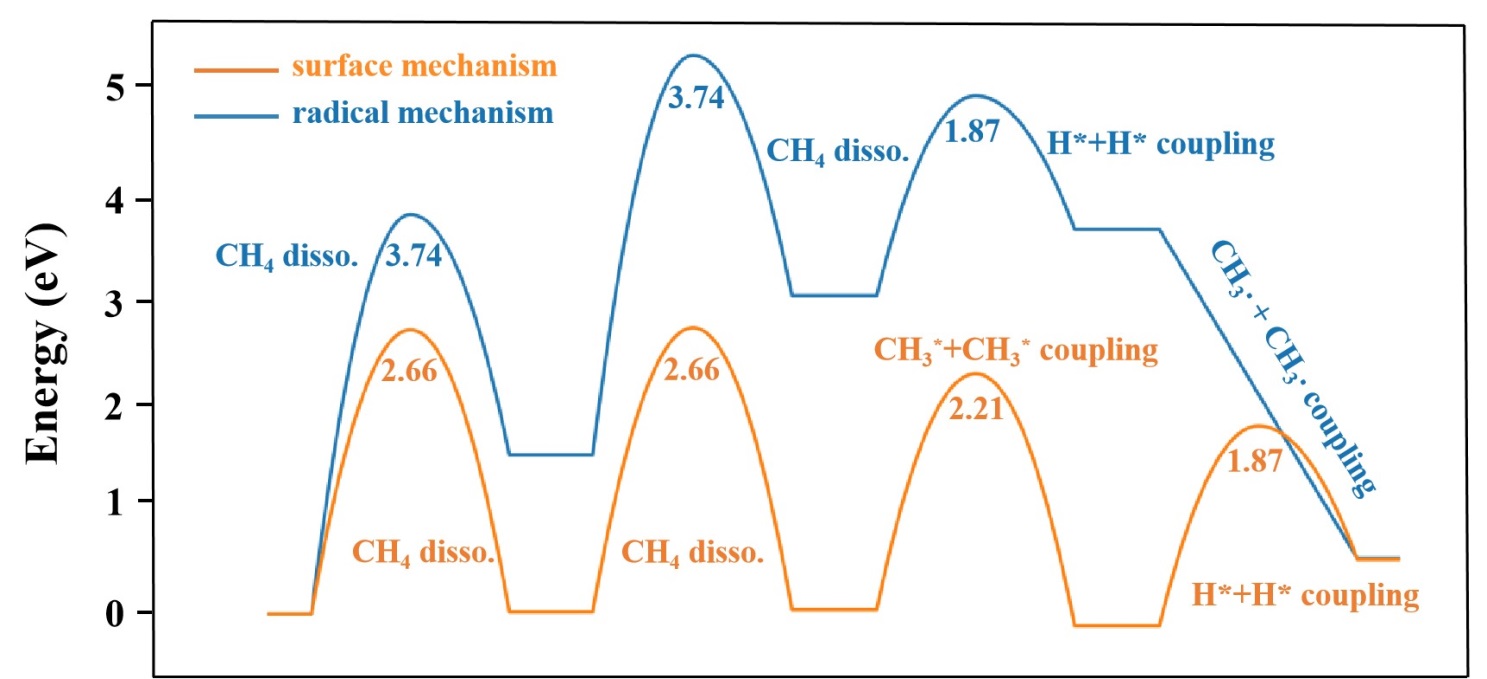


# Supplementary Fig. 38 | The free energy diagrams (at 750 ℃) of CH_4_ conversion towards CH_3_CH_3_(g) on Mo_2_C{101}, which shows that the surface mechanism is more favored than the methyl radical pathway.


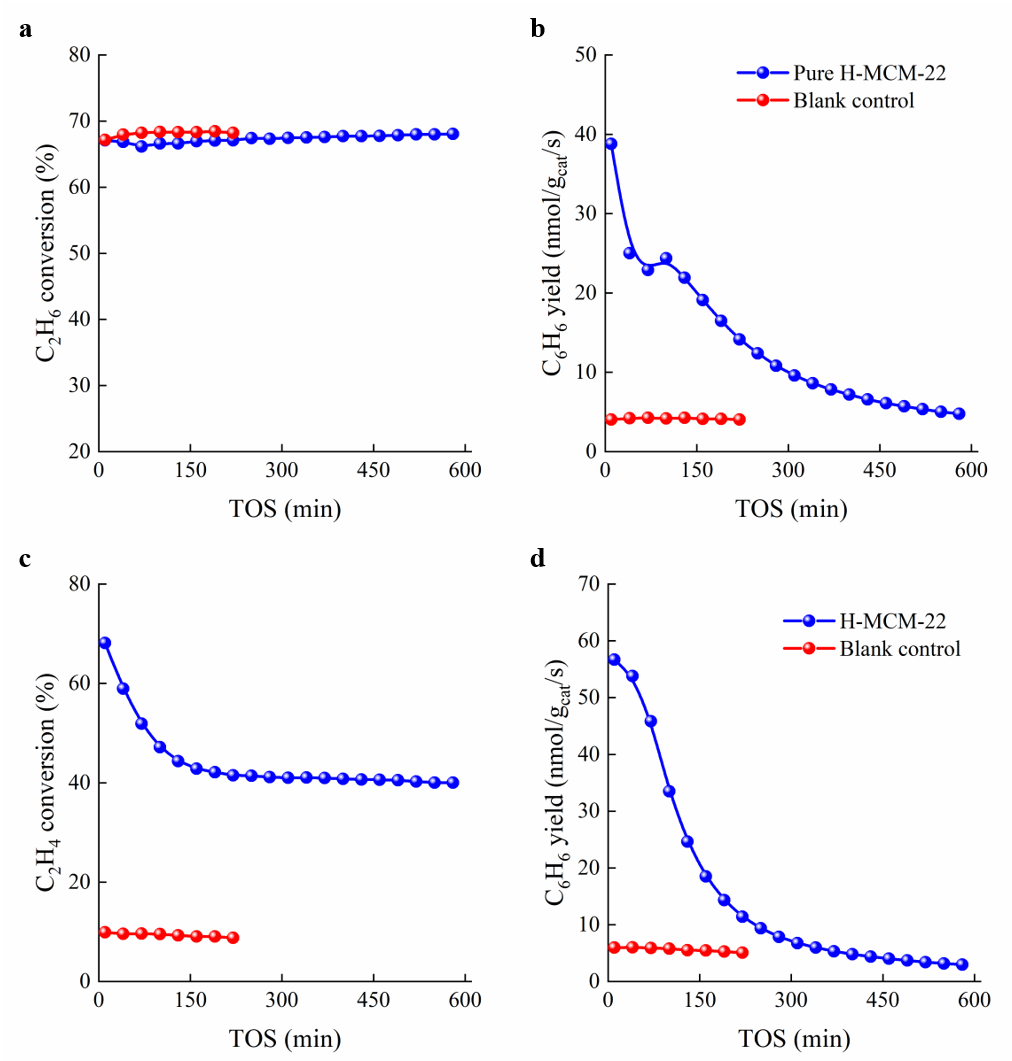


# Supplementary Fig. 39 | **a**, **b**, Ethane aromatization over pure H-MCM-22. Reaction conditions: 750 ℃, 0.1 MPa, C_2_H_6_:N_2_ = 1:9, and 1.5 L/(g_cat_·h). **c**, **d**, Ethylene aromatization over pure H-MCM-22. Reaction conditions: 750 ℃, 0.1 MPa, C_2_H_4_:N_2_ = 1:9, and 1.5 L/(g_cat_·h).


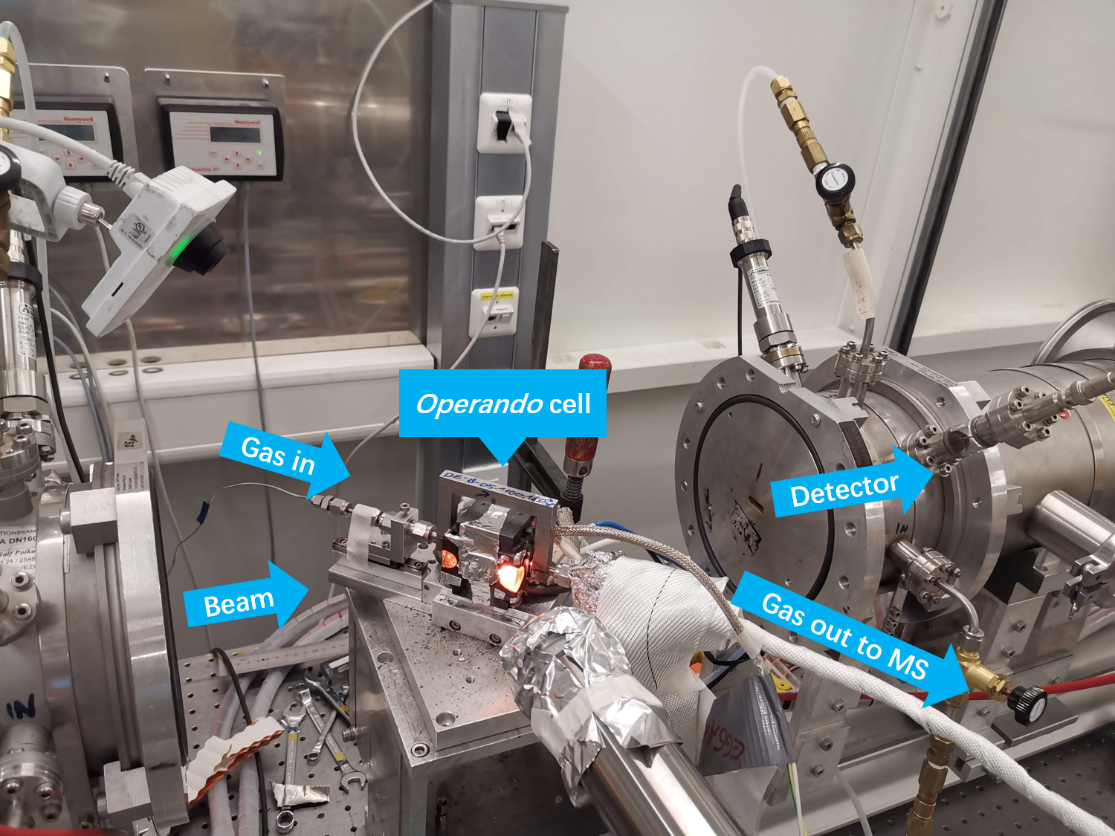


# Supplementary Fig. 40 | View and key equipment of the setup used for the *operando* XAS measurement.


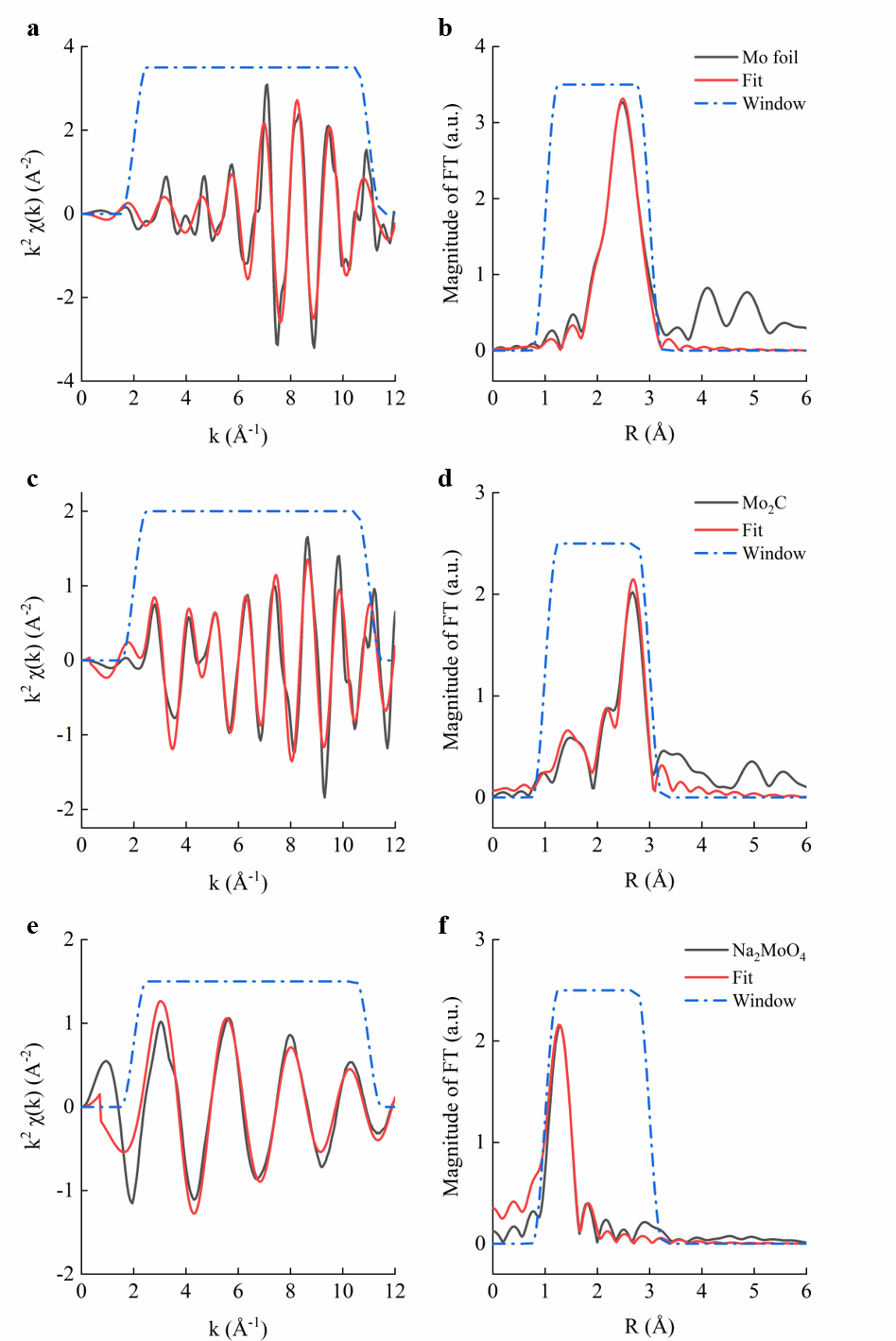


# Supplementary Fig. 41 | k^2^-weighted Mo K-edge EXAFS spectra and the corresponding Fourier transforms. **a, b**, Mo foil, **c, d**, Mo_2_C, and **e, f,** Na_2_MoO_4_.


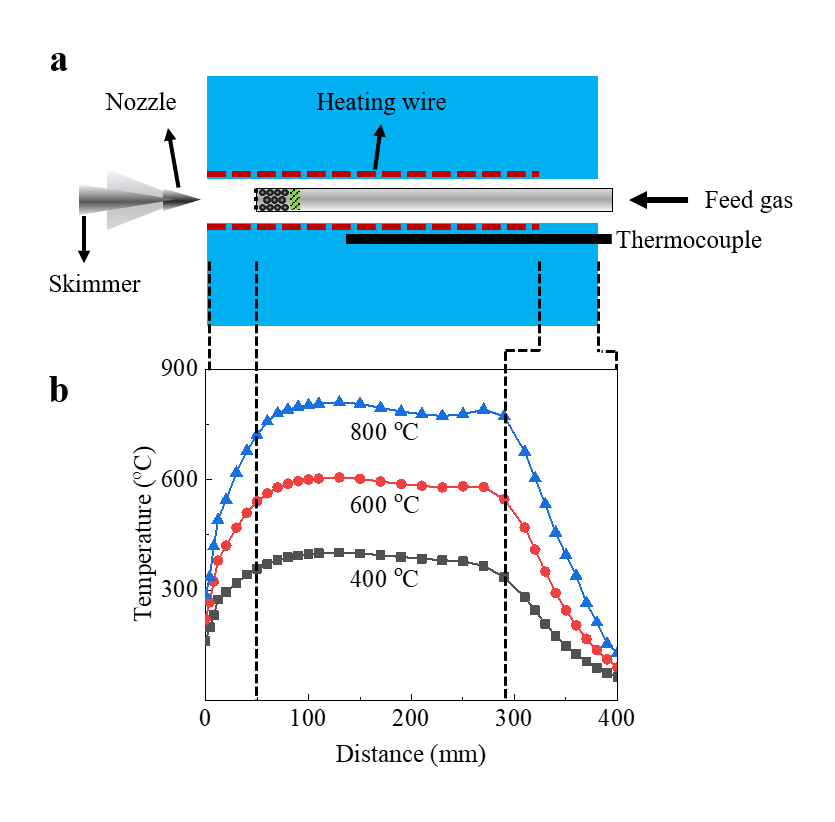


# Supplementary Fig. 42 | **a**, Schematic of the catalytic reactor with the SVUV-PIMS system^2^ and **b**, measured centerline temperature profiles.

# Supplementary Table 1 | ICP results of Mo-bearing catalysts.

| Samples | Mo loading | | Mo/Al mole ratio |
| --- | --- | --- | --- |
|  | (wt.%) | (μmol_Mo_/g_cat_) |  |
| 0.25Mo/MCM | 0.24 | 25.0 | 0.024 |
| 0.5Mo/MCM | 0.49 | 51.1 | 0.052 |
| 1Mo/MCM | 1.05 | 109.4 | 0.110 |
| 2Mo/MCM | 2.22 | 231.4 | 0.231 |
| 4Mo/MCM | 4.44 | 462.7 | 0.464 |
| 0.5Mo/SiO_2_^a^ | 0.53 | 55.2 | -- |
| Used 0.5Mo/SiO_2_^b^ | 0.53 | 55.2 | -- |
| 0.5Mo/ZSM | 0.53 | 55.2 | 0.041 |

^a^ The fresh catalyst in Supplementary Fig. 29;

^b^ The used catalyst in Supplementary Fig. 33 with the coke removed in 5 vol.%O_2_/Ar at 750 ℃ for 0.5 h. Note that the data indicate that there is negligible effect of MoO_3_ migration after reaction for 10 h under these conditions.

# Supplementary Table 2 | EXAFS fitting for 0.5Mo/MCM acquired EXAFS scan at 40 ℃ after He calcination and MDA reaction during *operando* XAS experiment.

| Samples | Path | R (Å) | CN | σ^2^ (10^-3^·Å^2^) | ΔE_0_ (eV) | R-factor^A^ (%) |
| --- | --- | --- | --- | --- | --- | --- |
| 0.5Mo/MCM(a.p.) | Mo-O_1_  Mo-O_3_ | 1.69 ± 0.02  1.89 ± 0.05 | 2.2 ± 0.3  1.2 ± 0.4 | 2*  2* | 4.1 ± 3.6 | 1.81 |
| 0.5Mo/MCM(He) | Mo-O_1_  Mo-O_2_ | 1.67 ± 0.01  1.79 ± 0.03 | 2.0 ± 0.5  2.0* | 2*  2* | 1.7 ± 2.3 | 0.85 |
| 0.5Mo/MCM(CH_4_) | Mo-O_2_  Mo-C/O_4_ | 1.81 ± 0.02  2.19 ± 0.04 | 2.1 ± 0.3  1.7 ± 0.7 | 2*  5* | 8.6 ± 2.9 | 3.14 |
| 0.5Mo/SiO_2_(a.p.) | Mo-O_1_  Mo-O_3_ | 1.71 ± 0.01  1.99 ± 0.02 | 1.1 ± 0.2  1.7 ± 0.2 | 2*  2* | 7.7 ± 3.1 | 6.14 |
| 0.5Mo/SiO_2_(He) | Mo-O_1_  Mo-O_3_ | 1.67 ± 0.01  1.97 ± 0.02 | 0.9 ± 0.2  2.0 ± 0.2 | 2*  2* | 5.1 ± 3.2 | 3.99 |
| 0.5Mo/SiO_2_(CH_4_) | Mo-O_1_  Mo-C  Mo-Mo_1_ | 1.67*  2.05 ± 0.02  2.80 ± 0.03 | 0.9 ± 0.2  2.7 ± 0.3  0.5* | 2*  5*  4* | 5.7 ± 1.9 | 4.36 |
| S_0_^2^ = 0.94; Fit range: 2 < k < 11, 1 < R < 3, 11 independent points  * Values were fixed to limit the number of fit parameters  ^A^ R-factor according to IFEFFIT^3^ | | | | | | |

# Supplementary Table 3 | EXAFS fitting for 0.5Mo/ZSM acquired EXAFS scan at 40 ℃ after MDA reaction during *operando* XAS experiment.

| Samples | Path | R (Å) | CN | σ^2^ (10^-3^·Å^2^) | ΔE_0_ (eV) | R-factor^A^ (%) |
| --- | --- | --- | --- | --- | --- | --- |
| 0.5Mo/ZSM(CH_4_) | Mo-O_2_  Mo-C/O_4_ | 1.75 ± 0.01  2.10 ± 0.03 | 1.3 ± 0.1  1.4 ± 0.3 | 2*  5* | 6.7 ± 2.3 | 3.21 |
| S_0_^2^ = 0.94; Fit range: 2 < k < 11, 1 < R < 3, 11 independent points  * Values were fixed to limit the number of fit parameters  ^A^ R-factor according to IFEFFIT^3^ | | | | | | |

# Supplementary Table 4 | Structural parameters extracted from the Mo K-edge EXAFS fitting.

| Samples | Path | R (Å) | CN | σ^2^ (10^-3^·Å^2^) | ΔE_0_ (eV) | R-factor^A^ (%) |
| --- | --- | --- | --- | --- | --- | --- |
| Mo foil | Mo-Mo_1_  Mo-Mo_3_ | 2.71 ± 0.01  3.13 ± 0.01 | 8*  6* | 3.8 ± 0.2  3.2 ± 0.4 | -1.1 ± 0.9 | 0.02 |
| Mo_2_C | Mo-C  Mo-Mo_2_ | 2.08 ± 0.02  2.98 ± 0.01 | 3*  12* | 5.3 ± 3.0  7.5 ± 0.5 | 0.4 ± 0.9 | 0.43 |
| Na_2_MoO_4_ | Mo-O_2_ | 1.78 ± 0.01 | 4* | 2.0 ± 0.7 | 2.0 ± 1.7 | 0.44 |
| S_0_^2^ = 0.94; Fit range: 2 < k < 11, 1 < R < 3, 11 independent points  * Values were fixed  ^A^ R-factor according to IFEFFIT^3^ | | | | | | |

# Supplementary References

1. Rehr, J. J. & Albers, R. C. Theoretical approaches to x-ray absorption fine structure. *Rev. Modern Phys.* **72**, 621-654 (2000).
2. Zhai, Y. et al. Experimental and kinetic modeling investigation on methyl decanoate pyrolysis at low and atmospheric pressures. *Fuel* **232**, 333-340 (2018).
3. Ravel, B. & Newville, M. ATHENA, ARTEMIS, HEPHAESTUS: data is for X-ray absorption spectroscopy using IFEFFIT. *J. Synchrotron Rad*. **12**, 537-541 (2005).
