# Supplementary material for: Methyl radical chemistry in non-oxidative methane activation over metal single sites
Source: Nat Commun. 2023 Sep 15;14:5716. doi: 10.1038/s41467-023-41192-y (PMC10504359; doi:10.1038/s41467-023-41192-y)
Supplement: Supplementary file 3 — Description of Additional Supplementary Files [file 41467_2023_41192_MOESM3_ESM.docx]

Description of Additional Supplementary Files

File Name: Supplementary Data 1

Description: Exemplary FEFF input file. XANES spectra at Mo k-edge of O=Mo-CH_2_ were simulated using the computer code FEFF 6.
