# Supplementary material for: Methyl radical chemistry in non-oxidative methane activation over metal single sites
Source: Nat Commun. 2023 Sep 15;14:5716. doi: 10.1038/s41467-023-41192-y (PMC10504359; doi:10.1038/s41467-023-41192-y)
Supplement: Supplementary file 4 — Supplementary Data 1 [file 41467_2023_41192_MOESM4_ESM.docx]

*Supplementary Data*

**Methyl radical chemistry in non-oxidative methane activation over metal single sites**

Xin Huang^1,†^, Daniel Eggart^2,†^, Gangqiang Qin^1,3,†^, Bidyut Bikash Sarma^4^, Abhijeet Gaur^2^, Jiuzhong Yang^5^, Yang Pan^5^, Mingrun Li^1^, Jianqi Hao^1^, Hongfei Yu^1^, Anna Zimina^4^, Xiaoguang Guo^1^, Jianping Xiao^1^, Jan-Dierk Grunwaldt^2,4,^*, Xiulian Pan^1,^*, Xinhe Bao^1,^*

^1^ State Key Laboratory of Catalysis, 2011-Collaborative Innovation Center of Chemistry for Energy Materials, Dalian Institute of Chemical Physics, Chinese Academy of Sciences, Dalian 116023, China

^2^ Institute for Chemical Technology and Polymer Chemistry, Karlsruhe Institute of Technology, Karlsruhe 76131, Germany

^3^ University of Chinese Academy of Sciences, Beijing 100049, China

^4^ Institute of Catalysis Research and Technology, Karlsruhe Institute of Technology, Eggenstein-Leopoldshafen 76344, Germany

^5^ National Synchrotron Radiation Laboratory, University of Science and Technology, Hefei 230029, China

^†^ These authors contributed equally: Xin Huang, Daniel Eggart, Gangqiang Qin

* Corresponding author. E-mail: grunwaldt@kit.edu; panxl@dicp.ac.cn; xhbao@dicp.ac.cn

**This file includes:**

# Exemplary FEFF input file

XANES spectra at Mo k-edge of O=Mo-CH_2_ were simulated using the computer code FEFF 6.

data_HCOAlSiMo

_audit_creation_method 'pos2cif.pl'

_cell_length_a 14.1145003795655

_cell_length_b 14.1145

_cell_length_c 24.8822

_cell_angle_alpha 90

_cell_angle_beta 90

_cell_angle_gamma 119.999999110424

_symmetry_space_group_H-M 'P1'

_symmetry_Int_Tables_number '1'

_symmetry_cell_setting 'triclinic'

loop_

_symmetry_equiv_pos_as_xyz

x,y,z

loop_

_atom_site_label

_atom_site_type_symbol

_atom_site_occupancy

_atom_site_fract_x

_atom_site_fract_y

_atom_site_fract_z

_atom_site_U_iso_or_equiv

H1 H 1.0000 0.51281 0.58773 0.51316 0.0000

H2 H 1.0000 0.39488 0.59458 0.48554 0.0000

C1 C 1.0000 0.47626 0.63930 0.50422 0.0000

O1 O 1.0000 0.36429 0.11607 0.13663 0.0000

O2 O 1.0000 0.36146 0.11397 0.31505 0.0000

O3 O 1.0000 0.89299 0.26904 0.12728 0.0000

O4 O 1.0000 0.90229 0.26971 0.31897 0.0000

O5 O 1.0000 0.75458 0.64434 0.13699 0.0000

O6 O 1.0000 0.75815 0.65123 0.31280 0.0000

O7 O 1.0000 0.57283 0.89377 0.12978 0.0000

O8 O 1.0000 0.57873 0.89808 0.31559 0.0000

O9 O 1.0000 0.11357 0.68812 0.12452 0.0000

O10 O 1.0000 0.12209 0.68611 0.30947 0.0000

O11 O 1.0000 0.32467 0.44188 0.13329 0.0000

O12 O 1.0000 0.32844 0.45120 0.30790 0.0000

O13 O 1.0000 0.10776 0.37892 0.86482 0.0000

O14 O 1.0000 0.12277 0.40537 0.67712 0.0000

O15 O 1.0000 0.25165 0.87852 0.85313 0.0000

O16 O 1.0000 0.27300 0.85419 0.70026 0.0000

O17 O 1.0000 0.62201 0.72764 0.86903 0.0000

O18 O 1.0000 0.58466 0.70429 0.67820 0.0000

O19 O 1.0000 0.87647 0.56404 0.86601 0.0000

O20 O 1.0000 0.85803 0.55238 0.69386 0.0000

O21 O 1.0000 0.68334 0.11365 0.87168 0.0000

O22 O 1.0000 0.70453 0.12410 0.69293 0.0000

O23 O 1.0000 0.41644 0.29804 0.87851 0.0000

O24 O 1.0000 0.40699 0.27188 0.69613 0.0000

O25 O 1.0000 0.60916 0.90525 0.86999 0.0000

O26 O 1.0000 0.62573 0.90960 0.69130 0.0000

O27 O 1.0000 0.08921 0.67968 0.87289 0.0000

O28 O 1.0000 0.06798 0.69930 0.67769 0.0000

O29 O 1.0000 0.29683 0.38867 0.86977 0.0000

O30 O 1.0000 0.28487 0.36672 0.69526 0.0000

O31 O 1.0000 0.36655 0.09107 0.86841 0.0000

O32 O 1.0000 0.34898 0.06286 0.67475 0.0000

O33 O 1.0000 0.89404 0.26194 0.85782 0.0000

O34 O 1.0000 0.90693 0.28481 0.67771 0.0000

O35 O 1.0000 0.72983 0.62486 0.85087 0.0000

O36 O 1.0000 0.71853 0.62671 0.68942 0.0000

O37 O 1.0000 0.90008 0.57946 0.12711 0.0000

O38 O 1.0000 0.90250 0.58485 0.30847 0.0000

O39 O 1.0000 0.69394 0.10418 0.14022 0.0000

O40 O 1.0000 0.69742 0.11455 0.30111 0.0000

O41 O 1.0000 0.41219 0.31962 0.12981 0.0000

O42 O 1.0000 0.41107 0.31853 0.31368 0.0000

O43 O 1.0000 0.11221 0.36896 0.13752 0.0000

O44 O 1.0000 0.11553 0.37442 0.30602 0.0000

O45 O 1.0000 0.25856 0.89824 0.14016 0.0000

O46 O 1.0000 0.26069 0.89926 0.31204 0.0000

O47 O 1.0000 0.63776 0.74833 0.13444 0.0000

O48 O 1.0000 0.64173 0.75038 0.31377 0.0000

O49 O 1.0000 0.27035 0.13376 0.50121 0.0000

O50 O 1.0000 0.84797 0.14499 0.49744 0.0000

O51 O 1.0000 0.83504 0.68954 0.50122 0.0000

O52 O 1.0000 0.72097 0.86089 0.50073 0.0000

O53 O 1.0000 0.17568 0.86714 0.49901 0.0000

O54 O 1.0000 0.15668 0.30502 0.49517 0.0000

O55 O 1.0000 0.68439 0.35918 0.99835 0.0000

O56 O 1.0000 0.31078 0.65319 0.00128 0.0000

O57 O 1.0000 0.47736 0.01419 0.14008 0.0000

O58 O 1.0000 0.48070 0.01584 0.31004 0.0000

O59 O 1.0000 0.00878 0.47853 0.15510 0.0000

O60 O 1.0000 0.00413 0.47728 0.28612 0.0000

O61 O 1.0000 0.53977 0.53302 0.14318 0.0000

O62 O 1.0000 0.54206 0.53177 0.30476 0.0000

O63 O 1.0000 0.99492 0.47570 0.83629 0.0000

O64 O 1.0000 0.00775 0.49453 0.70827 0.0000

O65 O 1.0000 0.46917 0.97608 0.85692 0.0000

O66 O 1.0000 0.48847 0.98947 0.69373 0.0000

O67 O 1.0000 0.51180 0.51206 0.85829 0.0000

O68 O 1.0000 0.50200 0.49098 0.69690 0.0000

O69 O 1.0000 0.65512 0.32726 0.27415 0.0000

O70 O 1.0000 0.34732 0.68501 0.27780 0.0000

O71 O 1.0000 0.35658 0.67849 0.72781 0.0000

O72 O 1.0000 0.63097 0.29486 0.72264 0.0000

O73 O 1.0000 0.53015 0.25453 0.07379 0.0000

O74 O 1.0000 0.55101 0.27228 0.18108 0.0000

O75 O 1.0000 0.54285 0.26365 0.36540 0.0000

O76 O 1.0000 0.35118 0.20994 0.40565 0.0000

O77 O 1.0000 0.72958 0.27927 0.08267 0.0000

O78 O 1.0000 0.74996 0.29420 0.18944 0.0000

O79 O 1.0000 0.74074 0.28843 0.36173 0.0000

O80 O 1.0000 0.78720 0.13833 0.39761 0.0000

O81 O 1.0000 0.69933 0.45351 0.08993 0.0000

O82 O 1.0000 0.72911 0.47250 0.19524 0.0000

O83 O 1.0000 0.71412 0.46218 0.35882 0.0000

O84 O 1.0000 0.86422 0.64940 0.40097 0.0000

O85 O 1.0000 0.45828 0.70370 0.07916 0.0000

O86 O 1.0000 0.45469 0.70858 0.18666 0.0000

O87 O 1.0000 0.45996 0.70861 0.36670 0.0000

O88 O 1.0000 0.65458 0.85742 0.40344 0.0000

O89 O 1.0000 0.31389 0.77352 0.08391 0.0000

O90 O 1.0000 0.28619 0.74659 0.19033 0.0000

O91 O 1.0000 0.30480 0.76324 0.36621 0.0000

O92 O 1.0000 0.14281 0.79354 0.39888 0.0000

O93 O 1.0000 0.24391 0.56132 0.09384 0.0000

O94 O 1.0000 0.25055 0.54087 0.19936 0.0000

O95 O 1.0000 0.24875 0.55396 0.36288 0.0000

O96 O 1.0000 0.20912 0.35742 0.39496 0.0000

O97 O 1.0000 0.27389 0.55098 0.91038 0.0000

O98 O 1.0000 0.27642 0.52845 0.80459 0.0000

O99 O 1.0000 0.32419 0.54940 0.64408 0.0000

O100 O 1.0000 0.21665 0.36370 0.59464 0.0000

O101 O 1.0000 0.28645 0.74621 0.91511 0.0000

O102 O 1.0000 0.25266 0.70504 0.80970 0.0000

O103 O 1.0000 0.23341 0.67536 0.64539 0.0000

O104 O 1.0000 0.20293 0.82603 0.59932 0.0000

O105 O 1.0000 0.46730 0.73245 0.92837 0.0000

O106 O 1.0000 0.45145 0.72770 0.82162 0.0000

O107 O 1.0000 0.44971 0.76486 0.63567 0.0000

O108 O 1.0000 0.64388 0.83838 0.59405 0.0000

O109 O 1.0000 0.67730 0.44228 0.90499 0.0000

O110 O 1.0000 0.70397 0.44973 0.79862 0.0000

O111 O 1.0000 0.66606 0.43719 0.64417 0.0000

O112 O 1.0000 0.81883 0.61475 0.59937 0.0000

O113 O 1.0000 0.76124 0.31038 0.91256 0.0000

O114 O 1.0000 0.74370 0.28431 0.80520 0.0000

O115 O 1.0000 0.74096 0.29916 0.63557 0.0000

O116 O 1.0000 0.79301 0.16077 0.59518 0.0000

O117 O 1.0000 0.54391 0.23065 0.92392 0.0000

O118 O 1.0000 0.53941 0.24528 0.81725 0.0000

O119 O 1.0000 0.52594 0.22644 0.62982 0.0000

O120 O 1.0000 0.32650 0.18589 0.60120 0.0000

O121 O 1.0000 0.36363 0.01415 0.22615 0.0000

O122 O 1.0000 0.20027 0.00022 0.41645 0.0000

O123 O 1.0000 0.98410 0.31764 0.22232 0.0000

O124 O 1.0000 0.99962 0.21915 0.41948 0.0000

O125 O 1.0000 0.65964 0.66317 0.22467 0.0000

O126 O 1.0000 0.80180 0.79481 0.42045 0.0000

O127 O 1.0000 0.57427 0.98335 0.22368 0.0000

O128 O 1.0000 0.86099 0.00572 0.42916 0.0000

O129 O 1.0000 0.01321 0.63077 0.21888 0.0000

O130 O 1.0000 0.00273 0.84367 0.44468 0.0000

O131 O 1.0000 0.41315 0.41317 0.22285 0.0000

O132 O 1.0000 0.14397 0.15271 0.42651 0.0000

O133 O 1.0000 0.01510 0.34115 0.76874 0.0000

O134 O 1.0000 0.00418 0.24133 0.57191 0.0000

O135 O 1.0000 0.37258 0.00948 0.77310 0.0000

O136 O 1.0000 0.16003 0.98560 0.57870 0.0000

O137 O 1.0000 0.60716 0.63986 0.77315 0.0000

O138 O 1.0000 0.83619 0.80939 0.58480 0.0000

O139 O 1.0000 0.99486 0.63261 0.77619 0.0000

O140 O 1.0000 0.00610 0.77772 0.56722 0.0000

O141 O 1.0000 0.61121 0.00681 0.77897 0.0000

O142 O 1.0000 0.86886 0.02577 0.57597 0.0000

O143 O 1.0000 0.39528 0.36885 0.78223 0.0000

O144 O 1.0000 0.13308 0.15456 0.56774 0.0000

O145 O 1.0000 0.46991 0.84305 0.49900 0.0000

Al1 Al 1.0000 0.78114 0.88886 0.56779 0.0000

Si1 Si 1.0000 0.58317 0.80276 0.34939 0.0000

Si2 Si 1.0000 0.57666 0.80422 0.65163 0.0000

Si3 Si 1.0000 0.66037 0.33575 0.06135 0.0000

Si4 Si 1.0000 0.67089 0.34158 0.20991 0.0000

Si5 Si 1.0000 0.66416 0.33600 0.33967 0.0000

Si6 Si 1.0000 0.33337 0.67380 0.06426 0.0000

Si7 Si 1.0000 0.33454 0.67051 0.21362 0.0000

Si8 Si 1.0000 0.33967 0.67689 0.34338 0.0000

Si9 Si 1.0000 0.33522 0.67110 0.93826 0.0000

Si10 Si 1.0000 0.33392 0.65966 0.79130 0.0000

Si11 Si 1.0000 0.33885 0.66568 0.66302 0.0000

Si12 Si 1.0000 0.66661 0.33450 0.93530 0.0000

Si13 Si 1.0000 0.65454 0.31921 0.78567 0.0000

Si14 Si 1.0000 0.64138 0.31464 0.65777 0.0000

Si15 Si 1.0000 0.46517 0.24117 0.13034 0.0000

Si16 Si 1.0000 0.41701 0.22639 0.34978 0.0000

Si17 Si 1.0000 0.24110 0.12402 0.43753 0.0000

Si18 Si 1.0000 0.76537 0.23604 0.13512 0.0000

Si19 Si 1.0000 0.78117 0.20275 0.34465 0.0000

Si20 Si 1.0000 0.87481 0.12795 0.43603 0.0000

Si21 Si 1.0000 0.77181 0.53815 0.13733 0.0000

Si22 Si 1.0000 0.80909 0.58677 0.34501 0.0000

Si23 Si 1.0000 0.87638 0.74373 0.44212 0.0000

Si24 Si 1.0000 0.53099 0.76429 0.13275 0.0000

Si25 Si 1.0000 0.24183 0.77578 0.13492 0.0000

Si26 Si 1.0000 0.20798 0.78684 0.34662 0.0000

Si27 Si 1.0000 0.13081 0.87600 0.43986 0.0000

Si28 Si 1.0000 0.23328 0.47732 0.14129 0.0000

Si29 Si 1.0000 0.22424 0.43312 0.34306 0.0000

Si30 Si 1.0000 0.12673 0.25811 0.43399 0.0000

Si31 Si 1.0000 0.23875 0.46193 0.86234 0.0000

Si32 Si 1.0000 0.23593 0.42019 0.65336 0.0000

Si33 Si 1.0000 0.12677 0.26552 0.55754 0.0000

Si34 Si 1.0000 0.22029 0.75230 0.86284 0.0000

Si35 Si 1.0000 0.19363 0.76456 0.65577 0.0000

Si36 Si 1.0000 0.13509 0.86417 0.56081 0.0000

Si37 Si 1.0000 0.53688 0.77310 0.87242 0.0000

Si38 Si 1.0000 0.74827 0.52037 0.85497 0.0000

Si39 Si 1.0000 0.76695 0.55978 0.65673 0.0000

Si40 Si 1.0000 0.87431 0.72614 0.56374 0.0000

Si41 Si 1.0000 0.77027 0.24178 0.86182 0.0000

Si42 Si 1.0000 0.78681 0.21689 0.65074 0.0000

Si43 Si 1.0000 0.87943 0.14112 0.56048 0.0000

Si44 Si 1.0000 0.46618 0.21706 0.87222 0.0000

Si45 Si 1.0000 0.40135 0.18630 0.65062 0.0000

Si46 Si 1.0000 0.22234 0.11413 0.56222 0.0000

Si47 Si 1.0000 0.36568 0.00993 0.16134 0.0000

Si48 Si 1.0000 0.36717 0.01029 0.29098 0.0000

Si49 Si 1.0000 0.99934 0.35927 0.16052 0.0000

Si50 Si 1.0000 0.00191 0.36152 0.28367 0.0000

Si51 Si 1.0000 0.64777 0.64739 0.16029 0.0000

Si52 Si 1.0000 0.64939 0.64800 0.28909 0.0000

Si53 Si 1.0000 0.57956 0.99939 0.15908 0.0000

Si54 Si 1.0000 0.58297 0.00357 0.28800 0.0000

Si55 Si 1.0000 0.00883 0.59328 0.15675 0.0000

Si56 Si 1.0000 0.01059 0.59468 0.28114 0.0000

Si57 Si 1.0000 0.42259 0.42755 0.15822 0.0000

Si58 Si 1.0000 0.42418 0.42883 0.28744 0.0000

Si59 Si 1.0000 0.00267 0.36504 0.83147 0.0000

Si60 Si 1.0000 0.01231 0.38185 0.70783 0.0000

Si61 Si 1.0000 0.36530 0.98857 0.83743 0.0000

Si62 Si 1.0000 0.37142 0.97918 0.71020 0.0000

Si63 Si 1.0000 0.61705 0.62500 0.83716 0.0000

Si64 Si 1.0000 0.60287 0.61375 0.70950 0.0000

Si65 Si 1.0000 0.98898 0.58734 0.83709 0.0000

Si66 Si 1.0000 0.98239 0.59425 0.71350 0.0000

Si67 Si 1.0000 0.59264 0.00107 0.84345 0.0000

Si68 Si 1.0000 0.60647 0.00765 0.71399 0.0000

Si69 Si 1.0000 0.40600 0.39231 0.84630 0.0000

Si70 Si 1.0000 0.39782 0.37582 0.71724 0.0000

Si71 Si 1.0000 0.76204 0.88027 0.43729 0.0000

Mo1 Mo 1.0000 0.54897 0.79137 0.51915 0.0000
